# Effect of Solution Stoichiometry on BaSO_4_ Crystallization from Turbidity Measurements and Modeling

**DOI:** 10.1021/acs.iecr.3c03612

**Published:** 2023-12-22

**Authors:** V. F. D. Peters, A. Baken, S. Y. M. H. Seepma, J. A. Koskamp, A. Fernández-Martínez, A. E. S. van Driessche, M. Wolthers

**Affiliations:** † Department of Earth Sciences, Utrecht University, Princetonlaan 8A, 3584 CB Utrecht, The Netherlands; ‡ Université Grenoble Alpes, Université Savoie Mont Blanc, CNRS, IRD, IFSTTAR, ISTerre, F-38000 Grenoble, France; § ESRF—The European Synchrotron, 71 Avenue des Martyrs, F-38000 Grenoble, France; ∥ Instituto Andaluz de Ciencias de la Tierra (IACT), CSIC—Universidad de Granada, Av. De las Palmeras 4, 18100 Armilla, Spain

## Abstract

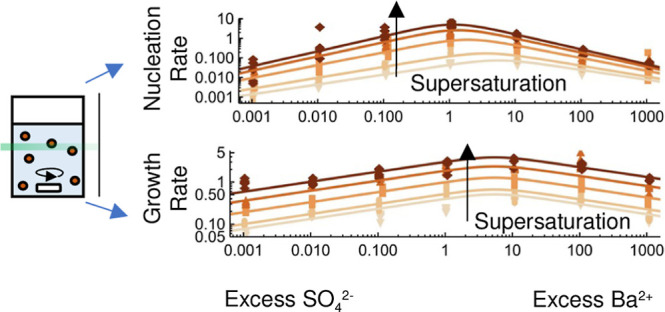

The impact of solution stoichiometry on the nucleation and growth of BaSO_4_ was studied by measuring solution transmittance and subsequent fitting to a crystallization model. Our results show that a large excess of either Ba^2+^ or SO_4_^2–^ ions inhibits both the nucleation and growth of BaSO_4_. However, for a small excess of Ba^2+^, the growth is enhanced. The dependence of nucleation and growth rates on supersaturation and solution stoichiometry was captured by a semiempirical rate model. Hence, the solution stoichiometry is a highly relevant parameter while studying all aspects of BaSO_4_ crystallization, and it could be worthwhile to examine other minerals similarly.

## Introduction

Understanding BaSO_4_ (Barite) formation is of interest for geothermal energy production^1,2^ or climate studies of marine Barite^3−7^ among others. In an industrial setting, the formation of BaSO_4_ is problematic due to its low solubility and resulting scale formation in piping, while the formation of marine Barite is relevant for studying the history of ocean chemistry.^6,7^ As a result, considerable fundamental research has been done studying the nucleation and growth of BaSO_4_ and the influence of temperature, pH, salinity, and supersaturation.^8−15^ Often in these fundamental studies, the ratio between the Ba^2+^ and SO_4_^2–^ ion concentrations during BaSO_4_ formation equals the stoichiometry of the crystal structure. However, in most seawater, surface water, or groundwater, SO_4_^2–^ is present in large excess with Ba/SO_4_ concentration ratios ranging from 10^–6^ to 10^–2^,^16,17^ while in hydrothermal and engineered systems, either ion can be in excess with Ba/SO_4_ concentration ratios ranging from 10^–4^ to 10^3^.^18^

Previous studies on the effect of solution stoichiometry on BaSO_4_ precipitation^19−23^ indicated that it can influence the particle size, morphology, or induction time (here defined as the time between setting the supersaturation and observing new particles,^9^ which can vary depending on the type of measurement). From studying the growth of BaSO_4_ on a substrate for a range of ionic ratios using atomic force microscopy,^24,25^ it was revealed that the growth rate is reduced by orders of magnitude for strongly nonstoichiometric conditions, while growth can be stimulated by a small Ba^2+^ surplus. These results were explained using concepts of the AB-type crystal growth model,^26,27^ where the stoichiometric effect stems from the rate of attachment and detachment from the two ions to the surface.^28^ Recent experiments with dynamic light scattering (DLS) indicated that the ionic ratio also impacts the formation (nucleation) of BaSO_4_ particles.^22^ It was observed that BaSO_4_ nucleation is faster near stoichiometric conditions than nonstoichiometric conditions, but in contrast to the growth experiments,^24,25^ BaSO_4_ formation seemed faster in a SO_4_^2–^ surplus than a Ba^2+^ surplus.

The effect of stoichiometry on nucleation is intriguing, because the widely applied classical nucleation theory considers only one component (BaSO_4_ ion pair) and thus cannot factor in stoichiometry directly.^29^ Hence, our aim is to investigate how nucleation and growth are individually impacted by solution stoichiometry within a single experimental approach. To study the formation of new BaSO_4_ particles, we measured the solution light transmission over time. In these kinds of experiments, often the induction time is used as a metric to study the thermodynamics of particle nucleation.^30−32^ Particles need to grow to a certain size to become detectable, but this growth time is considered negligible compared to the waiting time for the first particles to form. To separate the effects of growth on the induction time, one needs some form of modeling.^29,33^ Previously, Dai et al.^14^ performed solution transmittance experiments during BaSO_4_ formation at stoichiometric conditions and varying levels of supersaturation. Instead of focusing primarily on the induction time, they proposed fitting the transmission data with a two-step crystallization model, which integrates models for nucleation, growth, aggregation, and scattering. Their results indicate that growth time is not a negligible part of the induction time. Therefore, we follow the approach of Dai et al.^14^ by fitting our measurements to a two-step crystallization model including all transmission data. Their model is extended to work for nonstoichiometric conditions. Our methodology allows us to systematically study a wide range of ionic ratios and supersaturation. Lastly, we fit the resulting nucleation and growth rates to obtain a semiempirical model that can describe the effects of both supersaturation and solution stoichiometry on nucleation and growth.

## Experimental Methods

Growth solutions were prepared by dissolving stock solutions of BaCl_2_·2H_2_O and K_2_SO_4_ salts in MilliQ water, as calculated by PHREEQC^34,35^ using the llnl database^36^ to obtain a range of supersaturation indices SI (1.7, 1.8, 2.05, 2.3, or 2.5) and stoichiometric ratios *r*_aq_ (0.001, 0.01, 0.1, 1, 10, 100, or 1000) upon mixing them. Here, SI is defined as
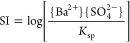
1where {Ba^2+^} and {SO_4_^2–^} are the activities of the respective ions, while *K*_sp_ is the solubility product (10^–10.09^ at 20 °C according to the llnl database^36^). The ionic ratio in solution is defined as the ratio between the activities of Ba^2+^ and SO_4_^2–^
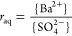
2

Additionally, KCl was added to the K_2_SO_4_ growth solution to obtain a final ionic strength of 0.2 mol L^–1^ after mixing, which is still below the ionic strength limit of the B-dot equation^37^ to calculate activities using the llnl database.^36,38^ A total ionic strength of 0.2 mol L^–1^ was used to ensure that it remained approximately constant during BaSO_4_ formation. The pH was predicted to be around 5.6, and this was also measured in the growth solutions before transmittance measurements. Note that while the pH and ionic strength remained constant in our batch experiments during the formation of BaSO_4_, the SI and *r*_aq_ values will change as Barite forms. Hence, we will refer to the initial values as SI_0_ and *r*_aq,0_. An overview of the solution compositions is found in Supporting Information 1. These conditions were chosen from preliminary experiments such that nucleation and growth occurred in a time frame between about 2 and 100 min, while the ionic strength should vary less than 5% in all cases in response to Barite formation. The choice of KCl as the background salt was motivated by preliminary experiments at *r*_aq,0_ = 1, where it showed faster BaSO_4_ formation kinetics than with NaCl. Additional preliminary measurements with NaCl at *r*_aq,0_ = 0.001 and *r*_aq,0_ = 1000 (see Supporting Information 2) indicated that this effect of background salt is rather small compared to the impact of stoichiometric ratio and supersaturation, in agreement with findings by Seepma et al.^23^

The growth solutions were filtered using a syringe with a 0.2 μm filter, before the Ba-containing and SO_4_-containing growth solutions were mixed. Hence, the possible presence of dust particles that could induce heterogeneous nucleation was avoided. In PMMA cuvettes, equal volumes (1 mL) of two growth solutions were mixed; one containing BaCl_2_ and the other containing K_2_SO_4_ and KCl. Using a Cary 50 UV–visible spectrophotometer, a laser light with a wavelength of 500 nm and a spectral bandwidth of 5.00 nm was passed through the cuvette, and the transmitted light was measured over time keeping the temperature constant at 20 °C. At the wavelength of 500 nm, the absorption of light is minimal^39^ and the transmitted light measured by UV–vis is related to the turbidity caused by the scattering of light by the particles,^30−32^ which in turn is related to the number density, shape, and size of newly formed particles. In order to avoid large local variations in supersaturation as much as possible, stirring was applied during the measurements, at a rate of 800 rpm. Each measurement was repeated six times in order to obtain reproducible results. After each measurement, the magnetic stirrer bars (length: 8 mm, diameter: 3 mm) were cleaned thoroughly with 10 mmol L^–1^ EDTA of pH 11 to remove the precipitated BaSO_4_, that is, the magnets were cleaned for at least 48 h, and EDTA was replaced a minimum of two times. This rigorous cleaning procedure was necessary to prevent secondary nucleation from occurring.

## Modeling

### Theory

The two-step crystallization model by Dai et al.^14^ combines aspects of scattering, nucleation, growth, and aggregation to explain changes in turbidity. Here, with aggregation, we mean the assemblage of smaller nucleated particles, which is more correctly referred to as agglomeration,^40^ but we preferred to use the same nomenclature reported by Dai et al.^14^ and related literature. In our model, we made specific adjustments to obtain a consistent fit for the different stoichiometries. In particular, the effect of aggregation could not be included explicitly as it would require mechanistic assumptions on how or if aggregation would be affected by solution stoichiometry. Additionally, including an aggregation rate constant in our model as a fit parameter led to too many fit parameters to obtain a unique fit. We have pointed out the differences in modeling throughout this section. The model assumes that crystallization occurs in two consecutive steps: nucleation and growth. At time *t*, for 0 < *t* ≤ *t*_n_, new nuclei are formed with a nucleation rate *J* and a critical radius *R*_c_. It is implied that the amount of growth during this nucleation step is negligible. Only after the nucleation time (*t* > *t*_n_), the nuclei are considered to grow to larger, detectable, particles, and it is assumed that no additional nuclei are formed. Hence, the model describes the mean crystallization behavior of the amount of particles that is determined in the nucleation step which grow to particles with a single size distribution in the growth step. While the model of Dai et al.^14^ uses the same approach, due to the inclusion of aggregative growth, a distribution of particles with different sizes is formed in the growth step.

### Scattering

The transmission of light is related to the scattering of light by the (growing) particles. Assuming light absorption is negligible, the turbidity is defined as^41^
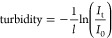
3where *I*_*t*_ and *I*_0_ are the transmitted and incident light intensity and *l* = 10 mm (size of cuvette) is the optical path length. Using the case of spherical particles with radius *R* and number density *n*, we relate the turbidity to particle properties by^41^
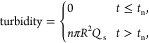
4where *Q*_s_ is the scattering efficiency factor. Using Mie scattering theory and Monte Carlo simulations, *Q*_s_ can be accurately described for spheres.^42,43^ Following these results, *Q*_s_ can be simplified to a polynomial expression for *R* ≲500 nm^14^
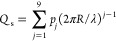
5where λ_0_ (=500 nm) is the wavelength and the constants *p*_*j*_ are given in Table 1 as stated in ref (14). These expressions also assume that there are no multiple scattering events. While this is likely true for the measurements at low SI_0_ and extreme *r*_aq,0_, multiple scattering events could affect the results at high turbidity (≳10 m^–1^) for high SI_0_ and near *r*_aq,0_ = 1, and a lower fit accuracy is expected at these conditions.^44^ While Dai et al.^14^ used the same scattering equations, the expression for *t* > *t*_n_ in eq 4 becomes a summation of all different particle sizes in their case.

**Table 1:** Fit Parameters to the Rate Models from eqs 10 and 13

ln *A*_*f*_	35.167 ± 0.289	ln *A*_α_	1.002 ± 0.074	ln *A*_β_	0.518 ± 0.186
*k*_*f*_	22.668 ± 1.119	*k*_α_	1.450 ± 0.280	*k*_β_	3.975 ± 0.711
ln *K*	–4.289 ± 0.041	α	0.239 ± 0.010	ln β	–1.694 ± 0.116

### Growth

The growth rate *G* = d*R*/d*t* determines how fast the radius *R* and as a consequence the turbidity increases over time. This is strongly correlated with the supersaturation. Since SI decreases over time as particles grow, *G* decreases as well. We describe this effect with a simple parabolic rate law^45^
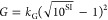
6where *k*_G_ is a fit parameter directly correlated with the growth rate. This expression and *k*_G_ can be connected to AB crystal growth models correlating the parameter *k*_G_ with kink formation and attachment and detachment frequencies of the ions.^26,46^ However, we use eq 6 to avoid making further assumptions on the growth mechanism.

We assume that SI does not decrease significantly during the nucleation step and that at the start of the growth step SI = SI_0_. As the critical radius *R*_c_ is only a few nm according to classical nucleation theory,^29^ the amount of formed BaSO_4_ should be negligible, making this assumption plausible. Using this assumption, we can express the change over time in SI by
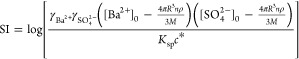
7where  and  are the initial concentrations in mol m^–3^, *R* is the radius of the growing particles in m, *n* is the number density of the growing spherical particles in no m^–3^, ρ = 4.48 × 10^3^ kg m^–3^ the mass density, *M* = 0.233 kg mol^–1^ the molar mass, and c* = 1 × 10^6^ mol^2^ m^–6^ is added for correct unit conversion of the concentrations. The same activity coefficients  or  were used for all measurements, as they were similar in all conditions calculated with the llnl database.^36^ Using eqs 6 and 7, it is not possible to obtain an analytical expression for *R*, and it needs to be solved numerically using Runge–Kutta methods with the additional condition *R*(*t*_n_) = *R*_c_.

According to the connections with the AB growth models, *k*_G_ is expected to be affected by *r*_aq_ and would therefore also change over time like SI.^26,46^ However, to avoid imposing an *r*_aq_ dependence beforehand, *k*_G_ is considered constant over time, so essentially *r*_aq_ = *r*_aq,0_ is assumed. The consequences of this assumption were checked by refitting the data to an expression of *G* with an explicit *r*_aq_ dependence (cf. eq 10) and confirming that there are no significant deviations (see Supporting Information 6).

In describing the growth, significant changes were made with respect to Dai et al.^14^ For the addition of ions to a particle, they employed a different expression based on a first order surface growth rate with *G* = ([Ba^2+^](*t*) – [Ba^2+^]_∞_)*k*_G_*M*/ρ, where [Ba^2+^](*t*) is the Ba^2+^ concentration in time (described as in eq 7) and  is the Ba^2+^ equilibrium concentration. However, this expression would not be valid for *r*_aq,0_ ≠ 1.^24,25^ Integrating this expression numerically leads to the size of the primary particle *R*_1_. Primary particles could aggregate to larger particles leading to a size distribution of particles  with size *R*_*j*_ = *j*^1/3^*R*_1_, where *j* indicates the amount of primary particles in the aggregate, *n*_0_ is the number of nuclei formed in the nucleation step, and τ = 1/(*k*_a_*n*_0_) is the characteristic time of aggregation. This, in turn, is related to an aggregation rate constant *k*_a_, which is fixed for Brownian aggregation.

### Fitting Procedure

In order to fit the model to the data from the measurements, the raw data were processed as follows. The raw output log *I*_0_/*I*_*t*_ was normalized to the average value of the initial signal, before the signal starts to increase due to scattering. Then, it was converted to turbidity using eq 3. The data were cutoff at *t*_c_ when they reached their maximum value. This was done because at high supersaturation, the turbidity started to decrease slowly after reaching a maximum due to sedimentation. As this behavior is not taken into account in the model, a more consistent fitting was found by cutting off this last part of the measurement.

The results of each separate measurement could, in principle, be fitted to eq 4 using four fitting parameters: *t*_n_, *n*, *k*_G_, and *R*_c_. However, this did not lead to consistent unique fits. According to classical nucleation theory, *R*_c_ is expected to be only a few nm at our supersaturations.^29^ However, differences in such small values of *R*_c_ have a negligible impact on the fitted curves. The fit is influenced by *R*_c_ when it approaches 50 nm, but this is much larger than expected. Hence, we have instead used *R*(*t*_n_) = 0, leaving only three fitting parameters which lead to a consistent fit. Note that using, for example, *R*(*t*_n_) = 5 nm instead leads to minimal differences in the results, and *R*(*t*_n_) = 0 is merely used for simplification of the calculation. Our fitting procedure differs from that of Dai et al.^14^ They simultaneously fitted all measurements to their model, linking measurements at different SI_0_ with classical nucleation theory, instead of employing one fit for each separate measurement. We opted not to use their approach as it requires additional assumptions on how the fit parameters relate to SI and *r*_aq_, and it does not allow for visualization of the experimental spread between the duplicate measurements. Additionally, preliminary calculations more in line with their approach showed that the results were heavily influenced by outliers. Another difference is that Dai et al. cutoff their data for a turbidity higher than 10 m^–1^ to exclude multiple scattering events. In contrast, we chose to include the higher turbidity data, as excluding it resulted in nonunique fits that depended on the exact cutoff point. This was likely less problematic for Dai et al. due to their simultaneous fitting of all data within the constraints of classical nucleation theory.

As our fit involves nonlinear regression, we calculated the root-mean-square deviation (RMSD) instead of the typical *R*^2^ to indicate a goodness of our fit^47^
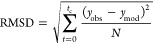
8where *y*_obs_ and *y*_mod_ represent, respectively, the measured and modeled value of the turbidity and *N*, the number of data points. This value can be interpreted as a deviation of the model with the experiments in units of turbidity. Since the values of turbidity can vary drastically for the different experimental conditions, RMSD can change a lot as well. To better compare how good the model works for different conditions, the normalized RMSD was calculated by dividing it over the entire range: NRMSD = RMSD/*y*_c_ × 100%, with *y*_c_ being the maximum turbidity at the cutoff.

To better interpret the effect of the fit parameters on the crystallization process, we calculated a nucleation rate *J* and initial growth rate *G*_0_ (at *t* = *t*_n_). To obtain *J* from the fitting parameters, we used *n*/*t*_n_ and for *G*_0_ we used eq 6 with SI = SI_0_ and the fitted *k*_G_. Subsequently, the rates were fitted to semiempirical models incorporating the effects of both SI and *r*_aq_. To obtain these rate models, we have combined different expressions capturing the dependence on SI and *r*_aq_, respectively. The fitting to the semiempirical rate models was done on a logarithmic scale and assuming a log-normal distribution of the rates due to the wide spread of *J* and *G*_0_ values depending on the experimental conditions. The SI dependence of the growth rate is already assumed in eq 6, but *k*_G_ is allowed to vary for different *r*_aq_. While this *r*_aq_ dependence can be connected to AB-type growth models,^26,46^ we have instead used a more general empirical model for ion-by-ion growth based on a Gaussian to avoid prior mechanistic assumptions^27^
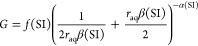
9where *f* relates to the maximum value, α relates to the width of the Gaussian, and β relates to the *r*_aq_-value at the maximum, where a maximum growth rate is reached at *r*_aq_ = 1/β. To combine eqs 6 and 9, we have taken the limits *r*_aq_ ≪ 1/β, *r*_aq_ = 1/β, and *r*_aq_ ≫ 1/β of both expressions and deduced the SI dependence of *f*, α, and β accordingly. This leads to the following combined expression
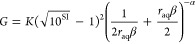
10where a new phenomenological parameter *K* is defined as a growth rate constant independent of *r*_aq_ and SI. It is related to *k*_G_ by
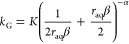
11

Note that in this combined expression, α and β are in fact not SI-dependent, and we used SI = SI_0_ and *r*_aq_ = *r*_aq,0_ to obtain *G*_0_. When compared again to mechanistic AB crystal growth models,^26,46^ parameters *K*, α, and β would be related to kink formation, attachment, and detachment frequencies.^27^ This exact expression was also used in recent modeling of AFM experimental data of BaSO_4_ growth on a substrate for SI ≲ 1 and varying *r*_aq_.^28^

For the SI dependence of the nucleation rate, we followed classical nucleation theory^29^ assuming that SI = SI_0_ and *r*_aq_ = *r*_aq,0_
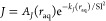
12where *A*_*J*_ and *k*_*J*_ are defined as phenomenological nucleation parameters with a dependency on *r*_aq_. Again, we kept the expression simple with parameters *A*_*J*_ and *k*_*J*_ as opposed to more elaborate expressions for these parameters which would imply more assumptions on the exact nucleation mechanism. However, *A*_*J*_ should still have a relation to the ion diffusion and the nucleation sites, whereas *k*_*J*_ should be strongly related to surface tension. It is possible that *A*_*J*_ has a dependency on SI as well due to its correlation with an incoming ion diffusion flux. However, the exact relation is unclear, and this can be hard to distinguish from experiments due to the strong exponential SI dependence in *J*, and therefore, it is not taken into account. We decided to use eq 9 for the *r*_aq_ dependence of *J* as it seemed to describe our results well. Using the same limits as before, we can deduce the following combined expression
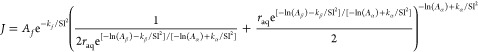
13with phenomenological parameters *A*_*f*_, *A*_α_, *A*_β_, *k*_*f*_, *k*_α_, and *k*_β_. These are more parameters than for eq 10 as now α and β do have an explicit SI dependence. The fitting procedure was performed in Wolfram Mathematica (see Supporting Information 3 for notebooks).

## Results and Discussion

### UV–Vis Measurements

**Figure 1:**
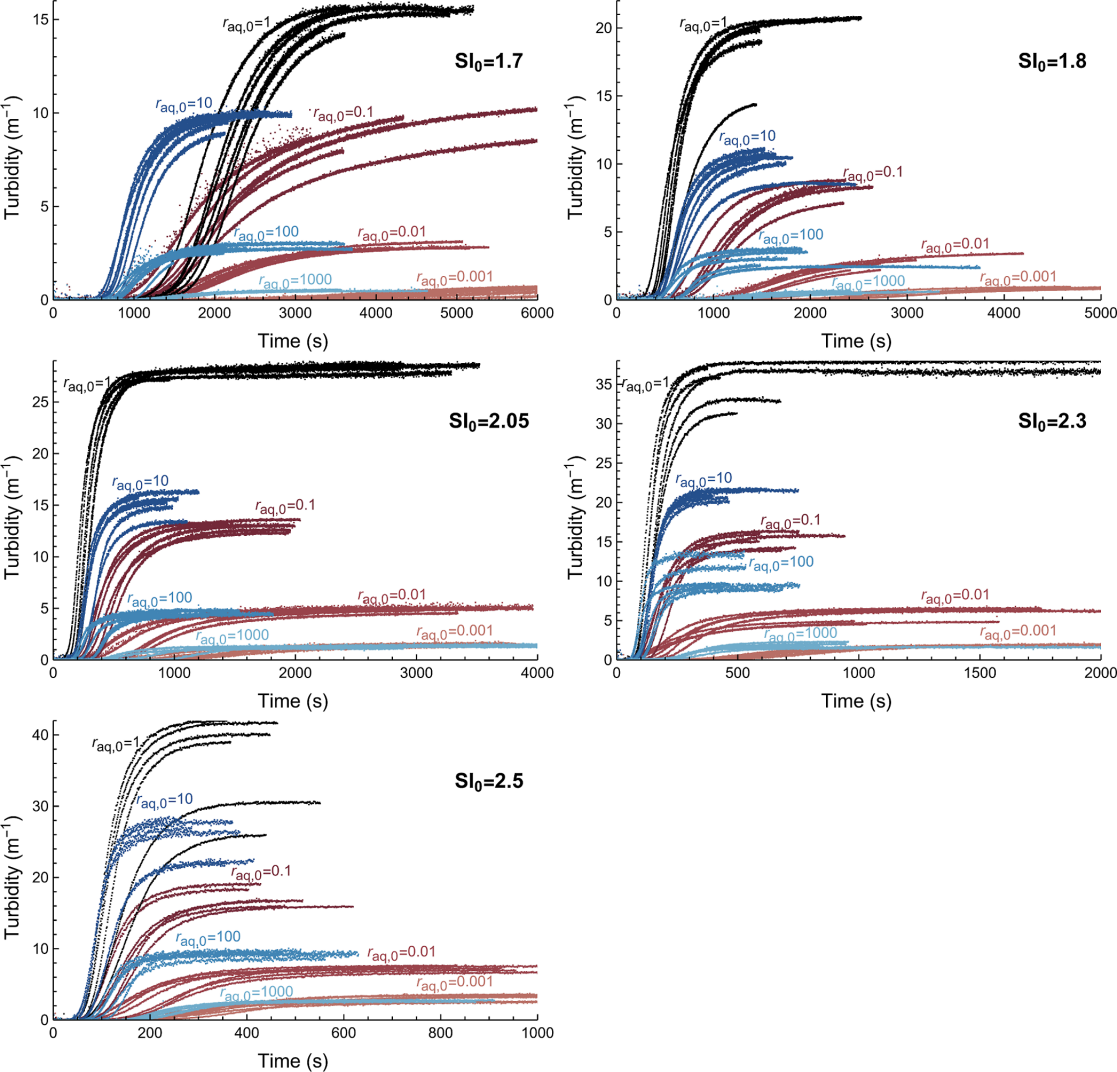
Turbidity evolution as a function of time for SI_0_ = 1.7, 1.8, 2.05, 2.3, and 2.5. Within each panel, 7 different data sets are plotted indicating different measurements at *r*_aq,0_ = 0.001, 0.01, 0.1, 1, 10, 100, and 1000. Note that in each panel, the axes have a different scale.

In Figure 1, the turbidity derived from the UV–vis measurements is shown for each initial supersaturation index SI_0_ and ionic ratio *r*_aq,0_. As the particles nucleate and grow, the turbidity increases due to the scattering of the laser light. At a certain moment, the turbidity reached a plateau value. A few anomalous measurements for the most extreme ratios (*r*_aq,0_ = 0.001, SI_0_ = 2.05 and *r*_aq,0_ = 1000, SI_0_ = 2.05) were left out of the graph and any further analysis because the turbidity was orders of magnitude higher than the other measurements at similar conditions (see Supporting Information 4). Additionally, for *r*_aq,0_ = 1 at all supersaturations and for *r*_aq,0_ = 0.1 and 100 at SI_0_ ≥ 2.05, the turbidity exceeds 10 m^–1^, suggesting the possibility of multiple scattering events.

Some trends related to *r*_aq,0_ are observed. The plateau value and overall magnitude of turbidity decrease strongly as *r*_aq,0_ deviates more from the ideal stoichiometric ratio of *r*_aq,0_ = 1. This means that less light is scattered in a strong surplus of either ion, which is expected due to the batch nature of the experiment. For the same SI_0_, a lot more material is formed at *r*_aq,0_ = 1 than for any other ratio. For example, at SI_0_ = 2.05 and *r*_aq,0_ = 1, around 0.32 mmol L^–1^ BaSO_4_ can be formed before equilibrium is reached, while for *r*_aq,0_ = 10 and the same SI_0_, only 0.15 mmol L^–1^ BaSO_4_ can form. Since the amount of formed BaSO_4_ correlates strongly with the scattered light and the turbidity, changes in magnitude of the turbidity for different *r*_aq,0_ are expected. For *r*_aq,0_ ≠ 1, it is expected that some of the surplus ions adsorb to the surface of crystal seeds since a surface charge was measured in these cases.^23^ This amount is likely still small compared to the overall material formed.

In most cases, it is also observed that the plateau is reached much later as *r*_aq,0_ deviates further from 1. This would indicate that crystallization (nucleation + growth) is slower in the surplus of either ion. Interestingly, for *r*_aq,0_ > 1 the plateau is always reached faster than for *r*_aq,0_ < 1 indicating that in a Ba^2+^ surplus crystallization is faster than in a SO_4_^2–^ surplus. From these experiments alone though, it is not clear to which degree the stoichiometry affects the nucleation or growth step of the crystal formation separately. Note that at each *r*_aq,0_, increasing SI_0_ generally leads to a higher plateau value and an overall increase in turbidity magnitude. Additionally, the plateau is reached faster (see Supporting Information 5 for separate graphs at each *r*_aq,0_ value).

### Fitting of Measurements

**Figure 2:**
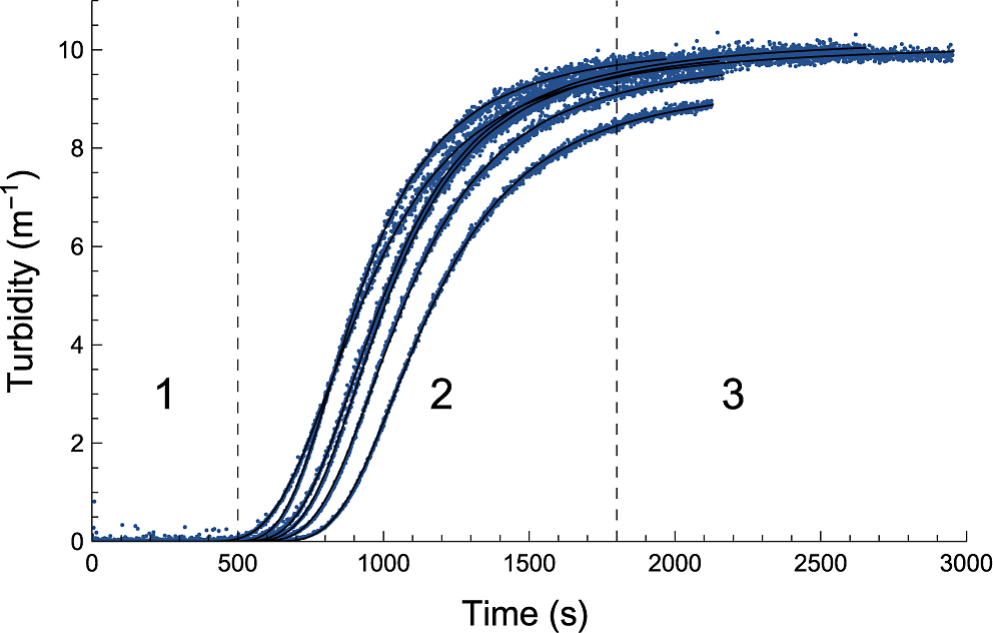
Example of fits of turbidity over time (solid curves) for six measurements (dotted series) at *r*_aq,0_ = 10 and SI_0_ = 1.7. The different regions of the curves indicate (1) no increase in turbidity, (2) turbidity increases due to growth, and (3) turbidity has (almost) reached a plateau value.

In Figure 2, six fits are shown as an example for *r*_aq,0_ = 10 and SI_0_ = 1.7, where the fitted curves are plotted through the data points. The curves fit the data well resulting in a normalized root-mean-square deviation (NRMSD) in turbidity between 0.5 and 1%. The graph is divided in three regions based on three characteristics, which are differently related to the three fitting parameters: (1) a period where there is no increase in turbidity, (2) a period where the turbidity increases due to growth, and (3) a period where the turbidity has (almost) reached a plateau value. For every measurement, all regions were fitted simultaneously, and it was noticeable that certain fitting parameters have a stronger influence in different regions and on the overall fit.

Region 1, which shows how long it takes before the particles become detectable, was influenced by all three fitting parameters. It depends on when the particles form (nucleation time *t*_n_), the amount of particles needed for detectability (number density *n*), and the rate of particles growth (growth rate parameter *k*_G_). Region 2, where the particles grow until the system approaches equilibrium, was primarily determined by *k*_G_ and *n* since *k*_G_ determines the rate of particle growth and *n* determines the total mass formed. In region 3, little growth is assumed as SI nears 0. Hence, the parameters related to the speed of crystallization, *t*_n_ and *k*_G_, do not strongly influence the turbidity found at this plateau, which was instead primarily determined by *n* and the initial concentrations. Note that the absolute value of *n* was quite sensitive to the initial concentrations, and therefore, it is expected to be more prone to error for the more extreme ratios *r*_aq,0_, where initial concentrations become increasingly smaller.

While unique solutions were obtained in all cases, it was noticed that the fit was more sensitive to certain parameters depending on how many regions it would affect. In particular, the sensitivity to *t*_n_ became problematic for *r*_aq,0_ = 0.001 and 0.01 and high SI_0_ (≥2.05) where region 1 was relatively short compared to region 2. The slow turbidity increase in region 2 implies a slow growth to a detectable size in region 1 and a short nucleation step (small *t*_n_). For 16 measurements in this parameter range (around 18% of all measurements at these conditions), this meant that the best fit would be found at *t*_n_ → 0, implying immediate nucleation, while often for a larger more realistic *t*_n_, the fit still looked acceptable visually. After refitting the measurements with eq 10, this was only the case for 8 measurements (see Supporting Information 6). Note that the refit showed the same overall trends discussed further on. However, it seems that a more refined crystallization model might be in order to describe these particular measurements accurately. In further analysis, all 16 measurements were excluded. It might have been possible to increase uniqueness by reducing the model to two fitting parameters as *k*_*R*_ and *t*_n_ seemed highly correlated, but this would have required implying some relation between the two parameters beforehand.

**Figure 3:**
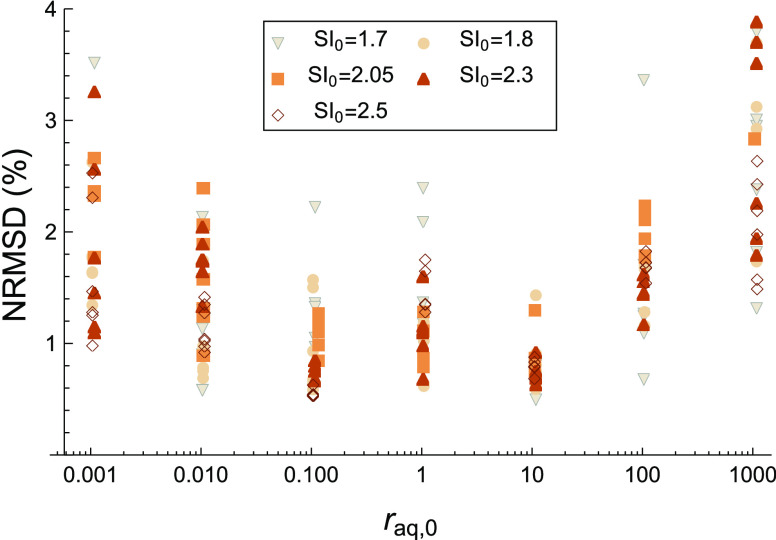
NRSMD is indicated for the fits of all different measurements.

To indicate how well the model fitted each measurement, we have plotted in Figure 3 the normalized root-mean-squared-deviation (NRMSD) in turbidity. This shows that in most cases, the error in the modeled and measured turbidity is around 1 to 3% of the maximum turbidity. Note that the NRMSD is influenced by both the experimental spread and by how well the model describes the experiment. Hence, for *r*_aq,0_ = 0.001 and 1000, and to a lesser extent *r*_aq,0_ = 0.01 and 100, where the turbidity is small and the relative experimental error is higher than that in the other cases, the NRMSD as a result is also higher than that of the other cases. This effect should be less pronounced at higher turbidity for 0.1 ≤ *r*_aq,0_ ≤ 10, and Figure 3 indicates that our model works best for *r*_aq,0_ = 10.

### Parameter Trends

**Figure 4:**
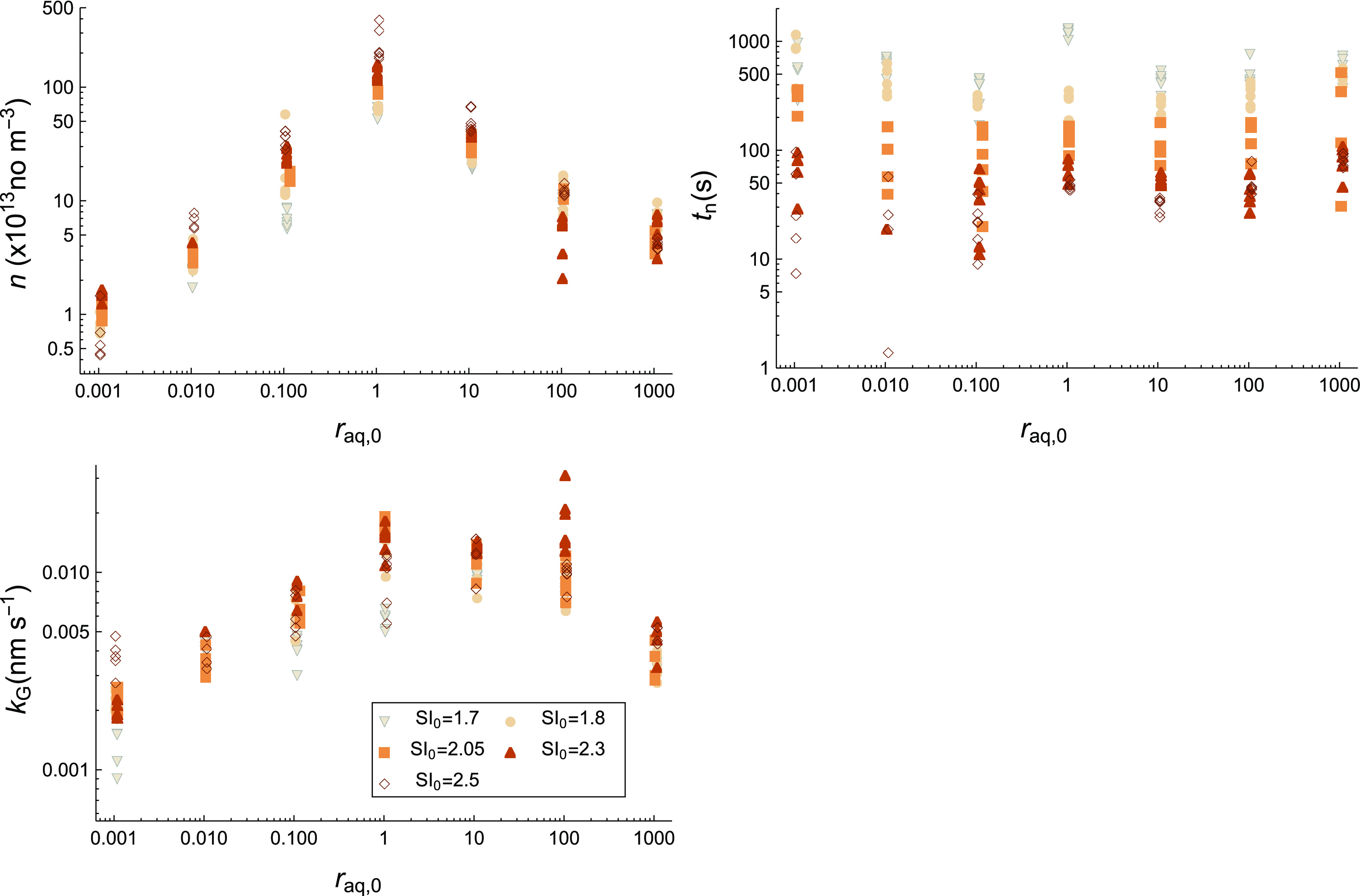
Number density of particles *n*, the nucleation time *t*_n_, and the growth parameter *k*_G_ is plotted as a function of *r*_aq,0_ for all SI_0_.

The fit parameters are plotted as a function of *r*_aq,0_ in Figure 4 for all different measurements at all SI_0_. It is important to note that all parameters are plotted on a log scale because the results of all the different measurements are better visualized.

Both *n* and *k*_G_ are strongly dependent on *r*_aq,0_. When there is a strong surplus of either ion (*r*_aq,0_ ≪ 1 or *r*_aq,0_ ≫ 1), both parameters decrease. The values decrease more steeply in a SO_4_^2–^ surplus (*r*_aq,0_ ≪ 1) compared to a Ba^2+^ surplus (*r*_aq,0_ ≫ 1). For *n*, there is a maximum around *r*_aq,0_ = 1, while for *k*_G_, the maximum value seems to be at a slight Ba^2+^ surplus depending on SI_0_ (between *r*_aq,0_ = 1 and *r*_aq,0_ = 100). The trends in these parameters capture the main trends discussed for Figure 1. The parameter *n* represents how much the plateau turbidity changes with respect to *r*_aq,0_ (taking the initial concentrations and thus the batch nature into account), while the parameter *k*_G_ correlates with how fast the turbidity increases to the plateau value. As a physical interpretation, this indicates that the solution stoichiometry can have a large effect on the amount of formed particles (and thus nucleation rate) and the growth rate.

When looking at these measurements for different SI_0_, the *n* and *k*_G_ values have a less consistent trend. For *n*, the effect of SI_0_ is different depending on *r*_aq,0_. A steady increase is seen when increasing SI_0_ for 0.01 < *r*_aq,0_ < 10, and this is consistent with what you would expect from classical nucleation, where the nucleation rate (proportional to *n*) is strongly dependent on SI_0_ (see eq 12). However, for more extreme ratios, the effect is inconsistent. Here, the *n* values remain constant or actually decrease a bit. Hence, it seems that SI_0_ becomes less relevant for the amount of formed particles as the ionic surplus is increased.

For the *k*_G_ values, their spread between different SI_0_ (at the same *r*_aq,0_) is a direct indication of how well eq 6 describes the actual growth of the particles. If eq 6 describes the SI dependence of the growth rate *G* exactly at one *r*_aq,0_ value, the *k*_G_ value remains constant between measurements at different SI_0_ for that same *r*_aq,0_ value. For most *r*_aq,0_, the value of *k*_G_ seems to increase slightly up to a certain maximum as SI_0_ increases (maximum at SI_0_ = 2.05, 2.3, or 2.5 depending on *r*_aq,0_), and it will then decrease slightly for higher SI_0_. This means that at lower SI_0_, the SI dependence on growth may be larger than eq 6 and at higher SI_0_, it may be smaller. This might be related to an increase in aggregation for higher SI_0_, but this is difficult to assess. Moreover, it seems that at *r*_aq,0_ = 1 and *r*_aq,0_ = 0.001, there is a rather large spread, indicating that our modeling works the least well at these conditions. At *r*_aq,0_ = 1, this could be attributed to a high concentration of small particles, multiple scattering, and/or a subtle shift in the crystallization mechanism leading to aggregation or nonclassical nucleation being more prominent.^48^ For *r*_aq,0_ = 0.001, this is likely related to a relatively high signal-to-noise ratio and sensitivity to concentration deviations. There is also a significant outlier at SI_0_ = 2.3 and *r*_aq,0_ = 100 possibly due to a shift in crystallization or morphology.^23^

For *t*_n_, a less consistent trend over *r*_aq,0_ is observed, and this varies for SI_0_. To better visualize the different trends depending on SI_0_ and *r*_aq,0_, we have included additional plots over SI_0_ in Supporting Information 7. For the *t*_n_ values, there is a more distinct correlation with SI_0_ instead of *r*_aq,0_, with a strong decrease in *t*_n_ as SI_0_ increases. This follows classical nucleation, where the nucleation rate (proportional to 1/*t*_n_) should strongly increase with SI_0_ (see eq 12). Consequently, at SI_0_ > 2.05, when the absolute values in *t*_n_ become small (<100s), there is no clear trend in nucleation time with *r*_aq,0_. At SI_0_ ≤ 2.05 and with a higher surplus of either ion (so from *r*_aq,0_ = 0.1 to *r*_aq,0_ = 0.001 or from *r*_aq,0_ = 10 to *r*_aq,0_ = 1000), *t*_n_ increases for most cases, indicating delayed nucleation. An exception is observed for SI_0_ = 1.7 and *r*_aq,0_ = 1, where the *t*_n_ values actually are higher than for a slight surplus. Overall, most of the trends of *t*_n_ over *r*_aq,0_ could be within error, and SI_0_ is evidently the more relevant factor for this parameter.

### Nucleation and Growth Rates

**Figure 5:**
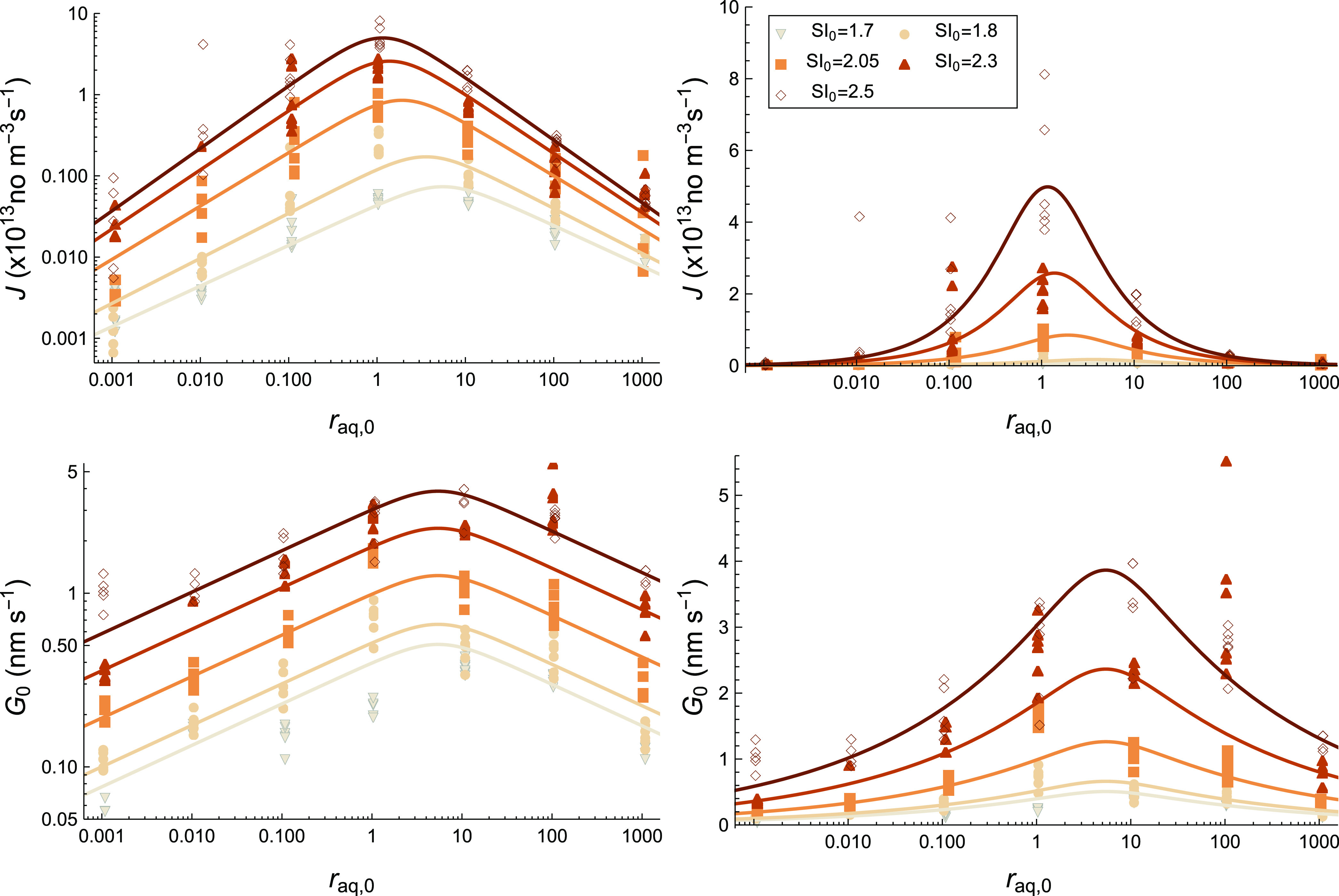
Nucleation rate *J* and initial growth rate *G*_0_ are plotted as data points over *r*_aq,0_. The curves represent the rate models from eqs 10 and 13 using the values listed in Table 1.

To examine the effect of the solution conditions on the speed of crystallization, the resulting nucleation (*J*) and initial growth (*G*_0_) rates are plotted as datapoints on both logarithmic and linear scales in Figure 5. The fitted models for the rates from eqs 10 and 13 are shown as curves with model parameters indicated in Table 1. Both rates are clearly influenced by supersaturation and solution stoichiometry. Because *J* (=*n*/*t*_n_) is both proportional to *n* and *t*_n_^–1^ and *n* has a large *r*_aq,0_ dependence, *J* also has a large *r*_aq,0_ dependence, despite *t*_n_ having a small *r*_aq,0_ dependence. It is the other way around for SI_0_ dependence, which is more pronounced for *t*_n_ than for *n*. The trends in *G*_0_ mirror those for *k*_G_ as it is the only fitting parameter that influences *G*_0_. This means that both rates reduce for high ionic surplus, and for *G*_0_, the maximum is shifted to a small surplus of Ba^2+^. Interestingly, for *J*, it seems that the *r*_aq,0_ dependence is different depending on SI_0_. For low SI_0_, it seems *J* is higher at a Ba^2+^-surplus compared to a SO_4_^2–^ surplus, while at higher SI_0_, the nucleation rate is more similar. Both *J* and *G*_0_ increase strongly as SI_0_ increases as expected, but *J* increases more strongly with SI_0_ for *r*_aq,0_ < 1 than *r*_aq,0_ > 1.

It is remarkable how the rate models (curves) still capture the overall trends as some changes in mechanisms or morphology are to be expected depending on the conditions.^20,22^ This implies that both classical nucleation and ion-by-ion growth at least partially describe the crystallization mechanism. Additionally, the Gaussian-like dependence on growth proposed by Hellevang et al.^27^ seems generally applicable for both nucleation and growth.

### Implications

It is not straightforward to compare our results to other experiments as different experimental methods cover different ranges in experimental conditions. Previously, both nucleation and growth of BaSO_4_ nanoparticles was studied by DLS during particle formation.^22,23^ However, the ionic strength was significantly lower (0.02 mol L^–1^ with NaCl as a background electrolyte) and supersaturation was higher with SI_0_ = 3. From DLS experiments under flow, it was deduced that nucleation becomes slower when *r*_aq,0_ ≠ 1, but with nucleation being more favorable at a SO_4_^2–^ surplus (*r*_aq,0_ < 1). This is in line with our nucleation rate model, which would predict a maximum nucleation rate at *r*_aq,0_ < 1 when extrapolated to SI_0_ = 3. Additionally, in the batch DLS experiments, almost immediate growth to particles of around 300 nm was observed which continued to grow with bulk growth rates of around 0.1 to 0.3 nm s^–1^ and a maximum growth rate at a small SO_4_^2–^ surplus. This contradicts our results for the initial growth rate as well as the results for growth on a substrate measured by atomic force microscopy,^24,25^ which show a maximum at a Ba^2+^ surplus and much larger values. However, for the batch DLS experiments, SI_0_ is significantly higher than those in either experiment. In the DLS batch experiments, particles formed with a size of around 300 nm before measurements even started, and the bulk growth rates were derived from the growth of these larger particles. It could be the case that supersaturation had already significantly decreased at this point leading to very different (lower) growth rates. Additionally, this suggests that at high SI_0_, there might be more than two steps in the crystallization mechanism with a fast (aggregative) growth to 300 nm particles and then a slower growth. The initial formation of an amorphous precursor phase, which could facilitate such an aggregative growth, has been suggested from titration and electron microscopy experiments at *r*_aq,0_ = 1.^48^

Due to the possibility of a more complex crystallization mechanism, it is worth discussing how this could affect the interpretation of our model results. Since the methodology of Dai et al.^14^ is very similar to ours, but they additionally account for aggregation, a comparison between our results at *r*_aq,0_ = 1 can give some insight. One notable difference should first be pointed out, though, in the background electrolyte, where they used NaCl to obtain an ionic strength of ∼1 mol L^–1^ instead of an ionic strength of 0.2 mol L^–1^ by adding KCl. Despite this different ionic strength, their measurements still occurred in a similar time frame for similar SI_0_. Because aggregation results in a reduction of particles *n* over time, we should compare the initial number density *n*_0_ of particles right after the nucleation step to estimate the influence on the nucleation parameters. Contrastingly, they have a larger difference between the number density *n*_0_ of initially formed particles for different SI_0_. Their *n*_0_ varied from 3 × 10^14^ to 5.5 × 10^17^ no m^–3^ for SI_0_ = 1.57 to SI_0_ = 2.60, while ours varied from 5.2 × 10^14^ to 3.1 × 10^15^ no m^–3^ for SI_0_ = 1.7 to SI_0_ = 2.5. Additionally, the nucleation times *t*_n_ are shorter leading with *J* = *n*_0_/*t*_n_ to nucleation rates *J* ranging from 3.8 × 10^11^ to 1.2 × 10^18^ no m^–3^ s^–1^ for Dai et al. as opposed to 4.4 × 10^11^ to 6.6 × 10^13^ no m^–3^ s^–1^ from our results. Hence, especially at high SI_0_ and a high amount of particles *n*, aggregation has the most prominent effect on the results. Since at *r*_aq,0_ ≠ 1, the amount of particles is smaller, it is expected that neglecting aggregation has a smaller impact on our results, and aggregation would not affect the overall trends in *J* over *r*_aq,0_. Additionally, as a surface charge was measured for *r*_aq,0_ ≠ 1,^23^ the particles might behave as charge-stabilized colloids preventing aggregation.^49^ If aggregation would then only impact *r*_aq,0_ = 1, a larger effect of solution stoichiometry is expected as the nucleation rate at *r*_aq,0_ = 1 would be higher. Again, this supports our assumption that neglecting aggregation would not affect the overall trends in *J* over *r*_aq,0_.

Further validation for excluding aggregation came from a preliminary fit of the data with a model that included aggregation (example in Supporting Information 8). This model overestimated turbidity via continued aggregation of larger particles as SI reaches 0, and better fits would only be obtained with a lower aggregation rate constant *k*_a_ (the limit of *k*_a_ → 0 leads to our proposed model). Experimental evidence supporting secondary aggregation of larger particles was also not observed in the aforementioned DLS measurements^22^ and accompanying transmission electron microscopy images. These images revealed only single crystals, twinning crystals, or small oriented aggregates depending on solution stoichiometry. Notably, these experiments were performed at lower ionic strength with less screening of the surface charge. Evidence for secondary aggregation has been observed at *r*_aq,0_ = 1.^50^

The ion-by-ion growth rate for Dai et al.^14^ is around a factor 10 lower than what we have observed. This is unsurprising since aggregation also contributes to particle growth in their model output, although the lower growth rate could also reflect the difference in background electrolyte and ionic strength. Hence, our growth rate *G*_0_ could be interpreted as an indication of the overall growth rate (ion-by-ion growth + aggregation). Again, since aggregation is expected to be less pronounced at *r*_aq,0_ ≠ 1 as less particles are formed, this would likely not change the trends observed in the growth rate for different *r*_aq,0_. Therefore, while the absolute values of *J* and *G*_0_ can be quite different depending on the exact mechanism assumed, the trends in the nucleation rate *J* and an overall growth rate *G* for different solution stoichiometry should remain similar to our results when aggregation would be taken into account. This was strengthened by the fact that the trends over SI_0_ and *r*_aq,0_ are captured by our rate models based on various mechanisms (curves Figure 5).

Due to its reliance on a two-step crystallization process involving classical nucleation theory and a bulk first order growth rate, the model of Dai et al.^14^ has been criticized.^51^ There,^51^ the authors advocate for a model including a four step mechanism based on slow, continuous nucleation followed by autocatalytic surface growth, bimolecular aggregation, and then secondary, size-dependent autocatalytic aggregation of smaller and larger particles. We have chosen our current approach, based on Dai et al.,^14^ as it provides a direct form of quantification of our type of measurements. Even within a four-step mechanism, the resulting fit parameters should still correlate with the formed number of particles (*n*), the delay time before a “burst” growth (*t*_n_), and a (composite) growth rate (*k*_G_). In turn, the significant changes in *n* and *k*_G_ for different solution stoichiometries are likely related to changes in initial particle formation (*n*) and a composite growth rate (*k*_G_).

We can speculate on why certain trends with *r*_aq,0_ are observed. When assuming that growth follows an AB-type growth mechanism, the growth behavior can be explained by considering the attachment and detachment rates.^27,28^ The attachment and detachment fluxes were derived from modeling isotope exchange experiments at near equilibrium conditions,^28^ and they also showed a smaller Ba^2+^ attachment flux than that for SO_4_^2–^ and a larger Ba^2+^ detachment flux. This in turn was related to differences in dehydration rates or surface complexation.^46^ To obtain more mechanistic insight on the stoichiometric effect on particle growth alone, one could also employ kinetic Monte Carlo calculations as previously used for particle dissolution.^52^ For nucleation, the formation of initial complexes might be relevant. To avoid highly charged particles, the amount of formed particles might be severely restrained to the concentration of the limiting ion. This would lead to much less particles *n* formed at *r*_aq,0_ ≠ 1 and thus a smaller nucleation rate *J*. For low SI_0_, critical nuclei are expected to be larger according to classical nucleation theory,^29^ and thus, the factors attributed to growth like attachment and detachment rate become more important.^53^ This might explain the shift of the maximum in *J* in Figure 5. Related to this could be the diffusion coefficients, as these were speculated to have an influence on the stoichiometric effect of CaSO_4_ formation.^54^

It is interesting to note that our results align with our earlier study of the effect of solution stoichiometry on CaCO_3_ formation.^55^ In that study, we observed that CaCO_3_ precipitation (nucleation + growth) was slower at nonstoichiometric ionic ratios for the same initial supersaturation, and this effect was reduced for higher supersaturations. Similar observations were made directly in the turbidity graphs for BaSO_4_ in Figure 1, and, for example, in the nucleation time *t*_n_ (Figure 4). Interestingly, CaCO_3_ precipitation was slower in a cationic excess with respect to an anionic excess, which is the opposite of what we had observed for BaSO_4_. Overall, this highlights the relevance of solution stoichiometry in the formation kinetics of these and likely most ionic crystals.

## Concluding Remarks

UV–vis measurements during the crystal formation of BaSO_4_ showed a strong effect of the initial ionic ratio on BaSO_4_ crystallization for the same degree of supersaturation. The measured signal could be fitted well to a model combining Mie scattering with two-step crystallization of nucleation and growth. This showed that a strong surplus of either Ba^2+^ or SO_4_^2–^ leads to a decrease in both the nucleation and growth rates compared to a stoichiometric ratio. For a SO_4_^2–^ surplus, the decrease in the growth rate was larger than that for a similar Ba^2+^ surplus. For a small Ba^2+^ surplus, the growth rate could even increase compared to a stoichiometric ratio. Regarding the nucleation rate, there was only a maximum at a Ba^2+^ surplus for low SI_0_. By combining expressions related to classical nucleation theory and AB crystal growth with that of a Gaussian, rate models could be obtained describing the dependence of the nucleation and growth rates on the degree of supersaturation and the ionic ratio over a wide range of supersaturation (1.7 ≤ SI ≤ 2.5) and solution ionic ratio (0.001 ≤ *r*_aq_ ≤ 1000). Overall, our methodology showed that the ionic ratio affects nucleation and growth for BaSO_4_ and will be of interest for examining other minerals as well.

## Supporting Information Available

Solution speciation for growth solutions, preliminary measurements with NaCl as background salt, example Wolfram Mathematica Notebooks for fitting procedure, additional turbidity measurements, alternative plots of turbidity measurements, refitting of measurements, alternative plots of *t*_n_, and example fit with aggregative growth (PDF)
