# Supplementary material for: Effect of Solution Stoichiometry on BaSO4 Crystallization from Turbidity Measurements and Modeling
Source: Ind Eng Chem Res. 2023 Dec 22;63(1):78–88. doi: 10.1021/acs.iecr.3c03612 (PMC10785810; doi:10.1021/acs.iecr.3c03612)
Supplement: Supplementary file 1 — ie3c03612_si_001.pdf [file ie3c03612_si_001.pdf]

The Effect of Solution Stoichiometry on $BaSO_4$ Crystallisation from Turbidity Measurements and Modeling

Supporting Information

V.F.D. Peters,[†] A. Baken,[‡,¶] S.Y.M.H. Seepma,[†] J.A. Koskamp,[†] A. Fernández-Martínez,[‡] A.E.S. van Driessche,[‡,§] and M. Wolthers[*,†]

†*Utrecht University, Department of Earth Sciences, Princetonlaan 8A, 3584 CB Utrecht, The Netherlands*

‡*Université Grenoble Alpes, Université Savoie Mont Blanc, CNRS, IRD, IFSTTAR, ISTerre, F-38000 Grenoble, France*

¶*ESRF – The European Synchrotron, 71 Avenue des Martyrs, F-38000 Grenoble, France*

§*Instituto Andaluz de Ciencias de la Tierra (IACT), CSIC – Universidad de Granada, Av. De las Palmeras 4, 18100 Armilla, Spain*

E-mail: m.wolthers@uu.nl

1. Solution Speciation

Table S1: Initial concentrations $[BaCl_2]_0$, $[K_2SO_4]_0$, $[KCl]_0$ are given in mmol/kg H_2O. $\{Ba^{2+}\}_0$, $\{SO_4^{2-}\}_0$, SI_0, $r_{aq,0}$, I, pH_c are as calculated by PHREEQC. pH_e was measured from the final growth solution.

$[BaCl_2]_0$	$[K_2SO_4]_0$	$[KCl]_0$	$\{Ba^{2+}\}_0$	$\{SO_4^{2-}\}_0$	SI_0	$r_{aq,0}$	I	pH_c	pH_e
0.007	10	175	2.10E-06	1.95E-03	1.70	0.001078	0.199	5.67	5.5
0.022	3.3	193	6.58E-06	6.37E-04	1.71	0.0103	0.201	5.65	5.5
0.07	1	198	2.10E-05	1.93E-04	1.69	0.109	0.200	5.64	5.2
0.22	0.33	200	6.58E-05	6.34E-05	1.71	1.04	0.201	5.63	5.3
0.7	0.1	199	2.09E-04	1.93E-05	1.69	10.9	0.201	5.63	5.4
2.2	0.033	194	6.59E-04	6.41E-06	1.71	103	0.200	5.63	5.4
7	0.01	180	2.10E-03	1.98E-06	1.71	1056.45	0.200	5.63	5.3
0.0077	11.5	173	2.31E-06	2.24E-03	1.80	0.00103	0.201	5.67	5.6
0.025	3.7	192	7.48E-06	7.14E-04	1.82	0.0105	0.200	5.65	5.6
0.077	1.15	198	2.30E-05	2.21E-04	1.80	0.104	0.200	5.64	5.6
0.25	0.37	199	7.48E-05	7.13E-05	1.82	1.049362	0.200	5.63	5.4
0.77	0.11	198	2.31E-04	2.13E-05	1.78	10.8	0.200	5.63	5.4
2.5	0.037	193	7.49E-04	7.20E-06	1.82	104	0.200	5.63	5.5
7.7	0.011	178	2.31E-03	2.19E-06	1.79	1050	0.200	5.63	5.4
0.0105	15	166	3.14E-06	2.92E-03	2.05	0.00108	0.202	5.69	5.6
0.033	4.85	190	9.87E-06	9.35E-04	2.05	0.0106	0.201	5.65	5.5
0.11	1.45	197	3.29E-05	2.79E-04	2.05	0.117824	0.200	5.64	5.6
0.33	0.49	198	9.89E-05	9.46E-05	2.06	1.044921	0.200	5.63	5.5
1.04	0.15	200	3.10E-04	2.88E-05	2.04	10.76816	0.203	5.63	5.7
3.3	0.048	191	9.88E-04	9.36E-06	2.05	105.4571	0.200	5.63	5.4
10.1	0.015	172	3.02E-03	3.01E-06	2.05	1003.658	0.202	5.63	
0.014	20	151	4.21E-06	3.94E-03	2.31	0.00107	0.200	5.70	5.5
0.044	6.6	184	1.32E-05	1.28E-03	2.32	0.0103	0.200	5.66	5.4
0.14	2	195	4.20E-05	3.86E-04	2.30	0.109	0.200	5.64	5.5
0.44	0.66	198	1.32E-04	1.27E-04	2.31	1.04	0.200	5.64	5.4
1.4	0.2	200	4.16E-04	3.83E-05	2.29	10.9	0.204	5.63	5.7
4.4	0.066	188	1.32E-03	1.29E-05	2.32	102	0.201	5.63	5.2
14	0.02	160	4.19E-03	4.09E-06	2.32	1020	0.201	5.63	5.3
0.017	25.3	138	5.12E-06	5.01E-03	2.50	0.00102	0.200	5.72	5.5
0.055	7.9	181	1.65E-05	1.53E-03	2.49	0.0108	0.200	5.66	5.5
0.17	2.53	194	5.09E-05	4.89E-04	2.48	0.104	0.200	5.64	5.6
0.55	0.79	198	1.64E-04	1.52E-04	2.49	1.08	0.201	5.64	5.7
1.7	0.25	195	5.09E-04	4.85E-05	2.48	10.5	0.200	5.63	5.4
5.5	0.079	185	1.64E-03	1.55E-05	2.50	106	0.201	5.63	5.3
17	0.024	150	5.10E-03	4.99E-06	2.49	1020	0.200	5.63	5.4

2. Preliminary Measurements with NaCl as Background Salt

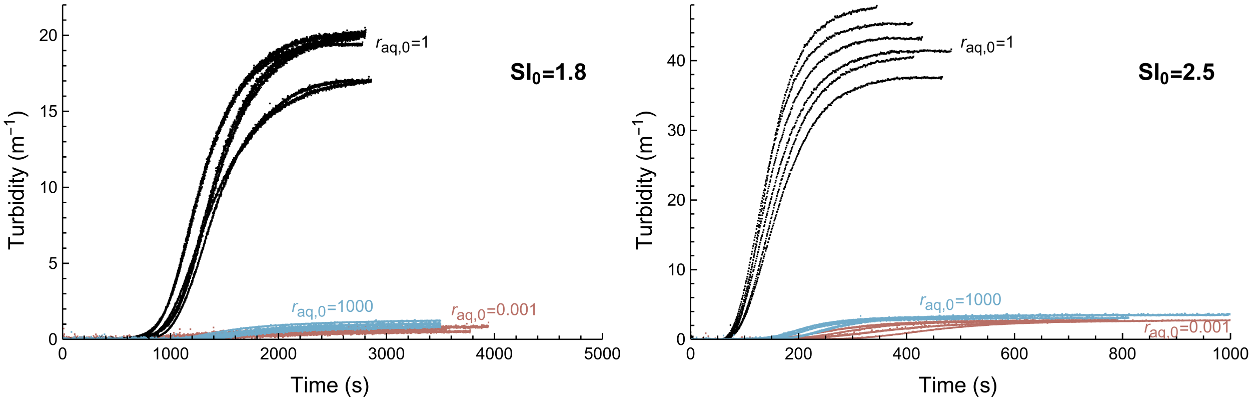

Figure S1: The turbidity is plotted over time for the measurements with 0.2 mol/L NaCl as background salt at SI_0=1.8 and SI_0=2.5 for $r_{aq,0}$ = 0.001, 1, or 1000. See Table S2 for the solution speciation.

Table S2: Initial concentrations $[BaCl_2]_0$, $[Na_2SO_4]_0$, $[NaCl]_0$ are given in mmol/kg H_2O. $\{Ba^{2+}\}_0$, $\{SO_4^{2-}\}_0$, SI_0, $r_{aq,0}$, I, pH_c are as calculated by PHREEQC. pH_e was measured from the final growth solution.

$[BaCl_2]_0$	$[Na_2SO_4]_0$	$[NaCl]_0$	$\{Ba^{2+}\}_0$	$\{SO_4^{2-}\}_0$	SI_0	$r_{aq,0}$	I	pH_c	pH_e
0.0077	11.3	175	2.31E-06	2.29E-03	1.81	0.00101	0.200	5.64	6.1
0.25	0.35	203	7.47E-05	7.00E-05	1.81	1.06684	0.201	5.60	5.6
7.7	0.011	180	2.31E-03	2.27E-06	1.81	1010	0.200	5.602	5.8
0.017	24.7	140	5.12E-06	5.08E-03	2.50	0.00101	0.200	5.69	6.1
0.55	0.79	200	1.65E-04	1.59E-04	2.51	1.04	0.200	5.60	5.6
17	0.023	152	5.10E-03	4.94E-06	2.49	1030	0.200	5.61	5.5

3. Example Wolfram Mathematica Notebooks for Fitting Procedure

Example notebooks are provided in addition to example data (`https://github.com/m-wolthers/Barite_Crystallisation_model_for_turbidity/tree/main`). Below are example notebooks for fitting a transmittance measurement and for fitting/plotting all trends.

Model Measurement

```
Remove["Global`*"]
SetDirectory[NotebookDirectory[]];
```

In[ ]:=

```
(*constants*)
lo = 10 * 10^-3 (*optical path length in m*);
λm = 500. * 10^-9 (*wavelength laser light in m*);
MW = 0.233 (*molar weight in kg mol^-1*);
ρp = 4.48 * 10^3 (*BaSO4 density in kg m^-3*);
kBa = 0.2993; kSO = 0.1934;(*activity coefficients Ba and SO4 ions*)
Ksp = 10^-10.09 * 10^6;
(*solubility product at 20 degrees Celsius * unit conversion factor*)

(*parameters scattering efficiency factor*)
p[1] = -1.52436718663 * 10^-3;
p[4] = 1.471719894 * 10^-1;
p[7] = -6.43936560641 * 10^-4;
p[2] = 1.95253706790 * 10^-2;
p[5] = -4.76815783236 * 10^-2;
p[8] = 2.36413082807 * 10^-5;
p[3] = -7.89209149687 * 10^-2;
p[6] = 7.62292501673 * 10^-3;
p[9] = -1.20408769568 * 10^-7;
```

In[ ]:=

```
(*defines turbidity function in m^-1*)
turbspn0[t_?NumericQ, n0i_?NumericQ, tni_?NumericQ, SI_?NumericQ,
  r_?NumericQ, kG_?NumericQ, max_?NumericQ] := (If[t ≤ tni, 0,
   (*numerical calculation of radius over time*)
   nr1[t, n0i, tni, SI, r, kG] =
    NDSolveValue[{aBa = kBa * (cBa[SI, r] - (4 π ρp n0i * 10^13 r1[tx]^3)/(3 MW)) ;
      aSO = kSO * (cSO[SI, r] - (4 π ρp n0i * 10^13 r1[tx]^3)/(3 MW));
      r1'[tx] == kG (((aBa * aSO)/Ksp)^(1/2) - 1)^2 * 10^-12, r1[tni] == 0},
     r1[t], {tx, tni, max}, Method → "ExplicitRungeKutta"];
   (*turbidity formula*)
   (Sum[p[j]/λm^(j-1) 2^(j-1) π^j nr1[t, n0i, tni, SI, r, kG]^(j+1) n0i * 10^13, {j, 1, 9}]) ])
```

Import + data processing

In[ ]:=
```
(*Note that the dataprocessing is specific for the format as in the exampledata file*)

(*Give SI0, raq0 and duplicate number of measurement to be fitted*)
SI = 2.5; r = 0.001; i = 4;
(*initial concentrations for the Ba and SO4 ions for [SI0,raq0]*)
cBa[SI, r] = 0.017(*mol m^-3*);
cSO[SI, r] = 25.3(*mol m^-3*);
tnorm[SI] = 20;(*Give normalisation region (in seconds) for[SI0]*)
```

In[ ]:=
```
(*imports measurement*)
AbsKCldatai[SI, r, i] = Import["exampledata.xlsx"];
AbsKCldatai[SI, r, i] =
  Select[AbsKCldatai[SI, r, i][[1, ;; , {1, 2}]], NumericQ[#[[1]]] &] /.
   {x_, z_} → {x / 1000, z}
   (*Selects relevant data and convert from miliseconds to seconds*);
```

In[ ]:=
```
(*Determine max cut-off absorbance*)
Absmax[SI, r, i] = Select[AbsKCldatai[SI, r, i][[tnorm[SI] ;;]],
    #[[2]] == Max[AbsKCldatai[SI, r, i][[tnorm[SI] ;;, 2]]] &, 1][[1]];
AbsKClnormi[SI, r, i] = AbsKCldatai[SI, r, i][[ ;; Absmax[SI, r, i][[1]]]] /.
   {x_, z_} → {x, z - Mean[Select[AbsKCldatai[SI, r, i], #[[1]] < tnorm[SI] &][[ ;; , 2]]]}
   (*Subtracts the normalisation value*);
turbKCli[SI, r, i] = AbsKClnormi[SI, r, i] /. {x_, z_} → {x, z/lo Log[10]}
  (*converts to turbidity*);
```

Fit

```
(*In the initial fit only number of particle n and nucleation time tn are
 fitted for a range of different kG values to find the minimum of RMSD*)
```

Initial fit

In[ ]:=
```
{n0start, tnstart} = {1, 200};(*Give initial estimate of
 number of particle n (× 10^13 no/m^3) and nucleation time tn (s)*)
{kGstart, bin, ntab} = {2.5, 0.25, 10};
(*Give lower range of growth parameter kG (× 10^-12 m/s),
step size for increasing kG values, and total number of calculated points*)
Module[{n0i = n0start, tni = tnstart, kG = kGstart},
 (*Plot fit of initial estimate as indication;
 change initial estimate if very different in magnitude*)
 max = turbKCli[SI, r, i][[-1, 1]];
 Show[ListPlot[turbKCli[SI, r, i], PlotRange → All],
  Plot[turbspn0[t, n0i, tni, SI, r, kG, max],
   {t, 0, turbKCli[SI, r, i][[-1, 1]]}, PlotRange → All]]]
```

```
In[ ]:= kGtable = {}; (*Creates a new table*)

        (*Fits turbidity function for ntab different values of kG increasing with bin*)
        For[kG = kGstart, kG < kGstart + ntab bin, kG = kG + bin,
          Print[kG];
          Monitor[fit = NonlinearModelFit[turbKCli[SI, r, i], {turbspn0[t, n0i, tni, SI, r, kG,
               max]}, {{n0i, n0start}, {tni, tnstart}}, t, AccuracyGoal → 1, PrecisionGoal → 1],
            {n0i, tni, kG}] (*fitting of turbidity function*);
          AppendTo[kGtable, ({n0i, tni, kG, Sqrt[fit["ANOVATableMeanSquares"]⟦-1⟧]} /.
              fit["BestFitParameters"]) (*create table of fitted values and RMSD*)];
           {n0start, tnstart} = kGtable⟦-1, ;; 2⟧
           (*use fitted n and tn values as initial values for the next fit*)];
        Clear[kG]

        ListPlot[kGtable⟦ ;; , {3, 4}⟧, PlotRange → All]
        (*Plot RMSD vs kG to check if minimum is within range;
        if not adjust kG range and calculate further*)

In[ ]:= Join[{{"n", "tn", "kG", "RMSD"}}, kGtable] // TableForm
```

Final fit

```
        {n0start, tnstart, kGstart} = Select[kGtable, #⟦4⟧ == Min[kGtable⟦ ;; , 4⟧] &]⟦1, ;; 3⟧
         (*Selects initial values from the values of the inital fit with the minimum RMSD*)

In[ ]:= Module[{n0i = n0start, tni = tnstart, kG = kGstart},
         (*Plot fit of initial estimate to ensure estimate is correct*)
         max = turbKCli[SI, r, i]⟦-1, 1⟧;
         Show[ListPlot[turbKCli[SI, r, i], PlotRange → All],
          Plot[turbspn0[t, n0i, tni, SI, r, kG, max],
            {t, 0, turbKCli[SI, r, i]⟦-1, 1⟧}, PlotRange → All]]]

        (*Fits all three parameters at once;
        if no convergence adjust AccuracyGoal/PrecisionGoal or accept the
         outcome as is with the kG table indicating the margin of error*)Monitor[
         fit = NonlinearModelFit[turbKCli[SI, r, i], {turbspn0[t, n0i, tni, SI, r, kG, max]},
            {{n0i, n0start}, {tni, tnstart}, {kG, kGstart}}, t,
            AccuracyGoal → 2, PrecisionGoal → 2], {n0i, tni, kG}]
        fit["BestFitParameters"]

        (*Forms a table of all relevant parameters for
         fitting (fitted parameters, RMSD, and max turbidity);
        note that other statistical parameters you could calculate
         might be unreliable if the final fit did not converge*)
        stats = {n0i, tni, kG, Sqrt[fit["ANOVATableMeanSquares"]⟦-1⟧],
            turbKCli[SI, r, i]⟦-1, 2⟧} /. fit["BestFitParameters"]
```

```
In[ ]:= Module[{n0i = stats⟦1⟧, tni = stats⟦2⟧, kG = stats⟦3⟧},
         (*Plot final fit to ensure it is correct*)
         max = turbKCli[SI, r, i]⟦-1, 1⟧;
         Show[ListPlot[turbKCli[SI, r, i], PlotRange → All],
          Plot[turbspn0[t, n0i, tni, SI, r, kG, max],
           {t, 0, turbKCli[SI, r, i]⟦-1, 1⟧}, PlotRange → All]]]

In[ ]:= Export["stats_r" <> ToString[r] <> "SI" <> ToString[SI] <> "i" <> ToString[i] <> ".xlsx",
          stats];(*Export all tabled stats to unique Excel
         file for this raq0, SI0, and duplicate number value*)
```

Model Trends

In[ ]:=
```
Remove["Global`*"]
SetDirectory[NotebookDirectory[]];
```

In[ ]:=
```
(*constants*)
kBa = 0.2993; kSO = 0.1934;(*activity coefficients Ba and SO4 ions*)
Ksp = 10^-10.09 * 10^6;
(*solubility product at 20 degrees Celsius * unit conversion factor*)
(*parameters scattering efficiency factor*)
```

In[ ]:=
```
SIc[SI_, r_] := Log10[1/Ksp kBa * (cBa[SI, r]) kSO * (cSO[SI, r])];
rac[SI_, r_] := (kBa * (cBa[SI, r]))/(kSO * (cSO[SI, r]));
```

Concentrations

In[ ]:=
```
(*concentrations for the Ba and SO4 ions for [SI0,raq0]*)
cBa[1.7, 0.001] = 0.007(*mol m^-3*);
cBa[1.8, 0.001] = 0.0077(*mol m^-3*);
cBa[2.05, 0.001] = 0.0105(*mol m^-3*);
cBa[2.3, 0.001] = 0.014(*mol m^-3*);
cBa[2.5, 0.001] = 0.017(*mol m^-3*);
cSO[1.7, 0.001] = 10(*mol m^-3*);
cSO[1.8, 0.001] = 11.5(*mol m^-3*);
cSO[2.05, 0.001] = 15(*mol m^-3*);
cSO[2.3, 0.001] = 20(*mol m^-3*); cSO[2.5, 0.001] = 25.3(*mol m^-3*);
cBa[1.7, 0.01] = 0.022(*mol m^-3*);
cBa[1.8, 0.01] = 0.025(*mol m^-3*);
cBa[2.05, 0.01] = 0.033(*mol m^-3*);
cBa[2.3, 0.01] = 0.044(*mol m^-3*); cBa[2.5, 0.01] = 0.055(*mol m^-3*);
cSO[1.7, 0.01] = 3.3(*mol m^-3*);
cSO[1.8, 0.01] = 3.7(*mol m^-3*);
cSO[2.05, 0.01] = 4.85(*mol m^-3*);
cSO[2.3, 0.01] = 6.6(*mol m^-3*); cSO[2.5, 0.01] = 7.9(*mol m^-3*);
cBa[1.7, 0.1] = 0.07(*mol m^-3*);
cBa[1.8, 0.1] = 0.077(*mol m^-3*);
cBa[2.05, 0.1] = 0.11(*mol m^-3*);
cBa[2.3, 0.1] = 0.14(*mol m^-3*); cBa[2.5, 0.1] = 0.17(*mol m^-3*);
cSO[1.7, 0.1] = 1(*mol m^-3*);
cSO[1.8, 0.1] = 1.15(*mol m^-3*);
cSO[2.05, 0.1] = 1.45(*mol m^-3*);
cSO[2.3, 0.1] = 2(*mol m^-3*); cSO[2.5, 0.1] = 2.53(*mol m^-3*);
cBa[1.7, 1] = 0.22(*mol m^-3*);
cBa[1.8, 1] = 0.25(*mol m^-3*);
cBa[2.05, 1] = 0.33(*mol m^-3*);
cBa[2.3, 1] = 0.44(*mol m^-3*); cBa[2.5, 1] = 0.55(*mol m^-3*);
```

```
cSO[1.7, 1] = 0.33(*mol m^-3*);
cSO[1.8, 1] = 0.37(*mol m^-3*);
cSO[2.05, 1] = 0.49(*mol m^-3*);
cSO[2.3, 1] = 0.66(*mol m^-3*); cSO[2.5, 1] = 0.79(*mol m^-3*);
cBa[1.7, 10] = 0.7(*mol m^-3*);
cBa[1.8, 10] = 0.77(*mol m^-3*);
cBa[2.05, 10] = 1.04(*mol m^-3*);
cBa[2.3, 10] = 1.4(*mol m^-3*); cBa[2.5, 10] = 1.7(*mol m^-3*);
cSO[1.7, 10] = 0.1(*mol m^-3*);
cSO[1.8, 10] = 0.11(*mol m^-3*);
cSO[2.05, 10] = 0.15(*mol m^-3*);
cSO[2.3, 10] = 0.2(*mol m^-3*); cSO[2.5, 10] = 0.25(*mol m^-3*);
cBa[1.7, 100] = 2.2(*mol m^-3*);
cBa[1.8, 100] = 2.5(*mol m^-3*);
cBa[2.05, 100] = 3.3(*mol m^-3*);
cBa[2.3, 100] = 4.4(*mol m^-3*); cBa[2.5, 100] = 5.5(*mol m^-3*);
cSO[1.7, 100] = 0.033(*mol m^-3*);
cSO[1.8, 100] = 0.037(*mol m^-3*);
cSO[2.05, 100] = 0.048(*mol m^-3*);
cSO[2.3, 100] = 0.066(*mol m^-3*); cSO[2.5, 100] = 0.079(*mol m^-3*);
cBa[1.7, 1000] = 7(*mol m^-3*);
cBa[1.8, 1000] = 7.7(*mol m^-3*);
cBa[2.05, 1000] = 10.1(*mol m^-3*);
cBa[2.3, 1000] = 14(*mol m^-3*); cBa[2.5, 1000] = 17(*mol m^-3*);
cSO[1.7, 1000] = 0.01(*mol m^-3*);
cSO[1.8, 1000] = 0.011(*mol m^-3*);
cSO[2.05, 1000] = 0.015(*mol m^-3*);
cSO[2.3, 1000] = 0.02(*mol m^-3*); cSO[2.5, 1000] = 0.024(*mol m^-3*);
```

Data Import + Processing

In[ ]:=
```
(*Imports all data from individual fits*)Do[fstats[SI, r] =
  Import["stats_r" <> ToString[r] <> "SI" <> ToString[SI] <> "*" <> ".xlsx"],
 {SI, {1.7, 1.8, 2.05, 2.3, 2.5}}, {r, {0.001, 0.01, 0.1, 1, 10, 100, 1000}}]

(*Alternative import from one combined datafile as 'fitdata.xlsx'*)
fitdata = Import["fitdata.xlsx"];
rangetab = Flatten[Table[{SI, r},
    {SI, {1.7, 1.8, 2.05, 2.3, 2.5}}, {r, {0.001, 0.01, 0.1, 1, 10, 100, 1000}}], 1];
(*Converts combined datafile to same format as original import*)
Do[fstats[rangetab⟦j, 1⟧, rangetab⟦j, 2⟧] =
  Table[{{{fitdata⟦j, i, 3⟧}, {fitdata⟦j, i, 4⟧}, {fitdata⟦j, i, 5⟧}, {fitdata⟦j, i, 6⟧},
     {fitdata⟦j, i, 7⟧}}}, {i, Length[fitdata⟦j⟧]}], {j, Length[rangetab]}]
```

In[ ]:=
```
(*Only takes the data with tn > 0*)
Do[fstatsRe[SI, r] = Select[fstats[SI, r], #⟦1, 2, 1⟧ > 0 &],
 {SI, {1.7, 1.8, 2.05, 2.3, 2.5}},
 {r, {0.001, 0.01, 0.1, 1, 10, 100, 1000}}]
```

In[ ]:=
```
(*Form tables of the relevant parameters*)
Do[NRMSDSI[SI] =
  Table[Table[{rac[SI, r], fstats[SI, r][[i, 1, 4, 1]] / fstats[SI, r][[i, 1, 5, 1]]}, {i, Length[fstats[SI, r]]}],
   {r, {0.001, 0.01, 0.1, 1, 10, 100, 1000}}],
 {SI, {1.7, 1.8, 2.05, 2.3, 2.5}}]
Do[datnSI[SI] = Flatten[Table[
    Table[{rac[SI, r], fstatsRe[SI, r][[i, 1, 1, 1]]}, {i, Length[fstatsRe[SI, r]]}],
    {r, {0.001, 0.01, 0.1, 1, 10, 100, 1000}}], 1],
 {SI, {1.7, 1.8, 2.05, 2.3, 2.5}}]
Do[dattnSI[SI] = Flatten[Table[
    Table[{rac[SI, r], fstatsRe[SI, r][[i, 1, 2, 1]]}, {i, Length[fstatsRe[SI, r]]}],
    {r, {0.001, 0.01, 0.1, 1, 10, 100, 1000}}], 1],
 {SI, {1.7, 1.8, 2.05, 2.3, 2.5}}]
Do[datkGSI[SI] = Flatten[Table[
    Table[{rac[SI, r], fstatsRe[SI, r][[i, 1, 3, 1]]}, {i, Length[fstatsRe[SI, r]]}],
    {r, {0.001, 0.01, 0.1, 1, 10, 100, 1000}}], 1],
 {SI, {1.7, 1.8, 2.05, 2.3, 2.5}}]
Do[datGSI[SI] =
  Flatten[Table[Table[{rac[SI, r], fstatsRe[SI, r][[i, 1, 3, 1]] (10^(SIc[SI,r]/2) - 1)^2},
     {i, Length[fstatsRe[SI, r]]}],
    {r, {0.001, 0.01, 0.1, 1, 10, 100, 1000}}], 1],
 {SI, {1.7, 1.8, 2.05, 2.3, 2.5}}]
datGall = Flatten[
   Table[Table[{SIc[SI, r], rac[SI, r], fstatsRe[SI, r][[i, 1, 3, 1]] (10^(SIc[SI,r]/2) - 1)^2},
     {i, Length[fstatsRe[SI, r]]}],
    {SI, {1.7, 1.8, 2.05, 2.3, 2.5}}, {r, {0.001, 0.01, 0.1, 1, 10, 100, 1000}}], 2];
Do[datJSI[SI] = Flatten[
   Table[Table[{rac[SI, r], fstatsRe[SI, r][[i, 1, 1, 1]] / fstatsRe[SI, r][[i, 1, 2, 1]]},
     {i, Length[fstatsRe[SI, r]]}],
    {r, {0.001, 0.01, 0.1, 1, 10, 100, 1000}}], 1],
 {SI, {1.7, 1.8, 2.05, 2.3, 2.5}}]
datJall = Flatten[Table[Table[{1 / SIc[SI, r]^2, rac[SI, r], fstatsRe[SI, r][[i, 1, 1, 1]] /
        fstatsRe[SI, r][[i, 1, 2, 1]]}, {i, Length[fstatsRe[SI, r]]}],
    {SI, {1.7, 1.8, 2.05, 2.3, 2.5}}, {r, {0.001, 0.01, 0.1, 1, 10, 100, 1000}}], 2];
```

Fits + Graphs

```
(*plotsettings*)colorscale = {ColorData["SiennaTones"][0.9],
   ColorData["SiennaTones"][0.7], ColorData["SiennaTones"][0.5],
   ColorData["SiennaTones"][0.3], ColorData["SiennaTones"][0]};
fstyle = FontSize → 8;
```

In[◦]:=
```
(*Plots NRMSD graph*)
error = ListLogLinearPlot[Table[Flatten[NRMSDSI[SI], 1] /. {x_, z_} → {x, 100 * z},
    {SI, {1.7, 1.8, 2.05, 2.3, 2.5}}], PlotRange → {0, 4},
   PlotStyle → colorscale, PlotMarkers → {▼, ●, ■, ▲, ◆}, PlotLegends →
    Placed[PointLegend[{"SI0=1.7", "SI0=1.8", "SI0=2.05", "SI0=2.3", "SI0=2.5"},
      LegendFunction → Framed, LabelStyle → 8, LegendMargins → 0,
      LegendMarkerSize → 5], {0.67, 0.75}], ImageSize → 300];
errorgrid = Grid[{{Rotate[Text["NRMSD (%)"], 90 Degree], error},
   {, Text[Style["raq,0", SingleLetterItalics → True]]}}]
```

In[◦]:=
```
(*Plots fitparameter graph*)
parpaper = Grid[{{Rotate[Text["n (x10^13no m^-3)"], 90 Degree],
    ListLogLogPlot[Table[datnSI[SI], {SI, {1.7, 1.8, 2.05, 2.3, 2.5}}],
     PlotRange → {{10^-3.2, 10^3.2}, {0.3, 500}}, PlotStyle → colorscale,
     PlotMarkers → {▼, ●, ■, ▲, ◆}, PlotLegends →
      Placed[PointLegend[{"SI0=1.7", "SI0=1.8", "SI0=2.05", "SI0=2.3", "SI0=2.5"},
        LegendFunction → Framed, LabelStyle → 8, LegendMargins → 0,
        LegendMarkerSize → 5], {0.1, 0.75}], ImageSize → 300],
    Rotate[Text["tn(s)"], 90 Degree],
    ListLogLogPlot[Table[dattnSI[SI], {SI, {1.7, 1.8, 2.05, 2.3, 2.5}}],
     PlotRange → {{10^-3.2, 10^3.2}, {1, 2000}}, PlotStyle → colorscale,
     PlotMarkers → {▼, ●, ■, ▲, ◆}, ImageSize → 300]},
   {, Text[Style["raq,0", SingleLetterItalics → True]], ,
    Text[Style["raq,0", SingleLetterItalics → True]]},
   {Rotate[Text["kG(nm s^-1)"], 90 Degree], ListLogLogPlot[
     Table[datkGSI[SI], {SI, {1.7, 1.8, 2.05, 2.3, 2.5}}] /. {x_, z_} → {x, z / 1000},
     PlotRange → {{10^-3.2, 10^3.2}, {0.00055, 0.035}}, PlotStyle → colorscale,
     PlotMarkers → {▼, ●, ■, ▲, ◆}, ImageSize → 300]},
   {, Text[Style["raq,0", SingleLetterItalics → True]]}}]

(*fit J on a log scale*)
fitJall = NonlinearModelFit[datJall /. {x_, y_, z_} → {x, y, Log[z] + Log[10^13]},
   lnAf - x kf - (lnAα - x kα) Log[r/2 Exp[-(lnAβ - kβ x)/(-lnAα + x kα)] + 1/(2 r) Exp[(lnAβ - kβ x)/(-lnAα + x kα)]],
   {{lnAf, 35}, {lnAα, 1}, {lnAβ, 0.7}, {kf, 26}, {kα, 2.1}, {kβ, 10}}, {x, r}];
Jparall = fitJall["ParameterTableEntries"][[;; , ;; 2]]


(*fit G0 on a log scale*)
fitGall = NonlinearModelFit[datGall /. {x_, y_, z_} → {x, y, Log[z / 1000]},
   lnK - α Log[(1/(2 r Exp[lnβ]) + (r Exp[lnβ])/2)] + Log[(10^(SI/2) - 1)^2],
   {{lnK, Log[0.01]}, {α, 0.2}, {lnβ, -Log[5]}}, {SI, r}];
Gparall = fitGall["ParameterTableEntries"][[;; , ;; 2]]
```

4. Additional Turbidity Measurements

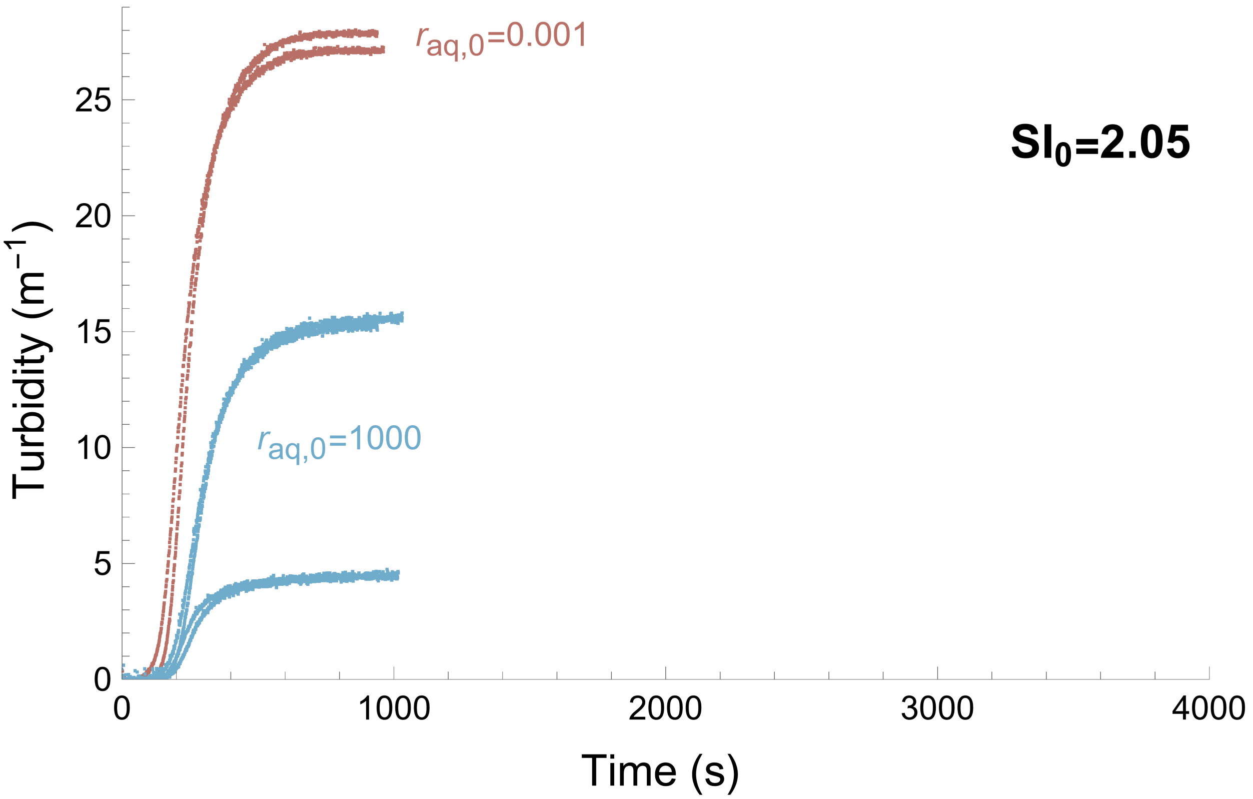

Figure S2: The turbidity is plotted over time for the anomalous measurements at SI$_0$=2.05 and $r_{\text{aq},0}$ = 0.001 or 1000.

5. Alternative Plots of Turbidity Measurements

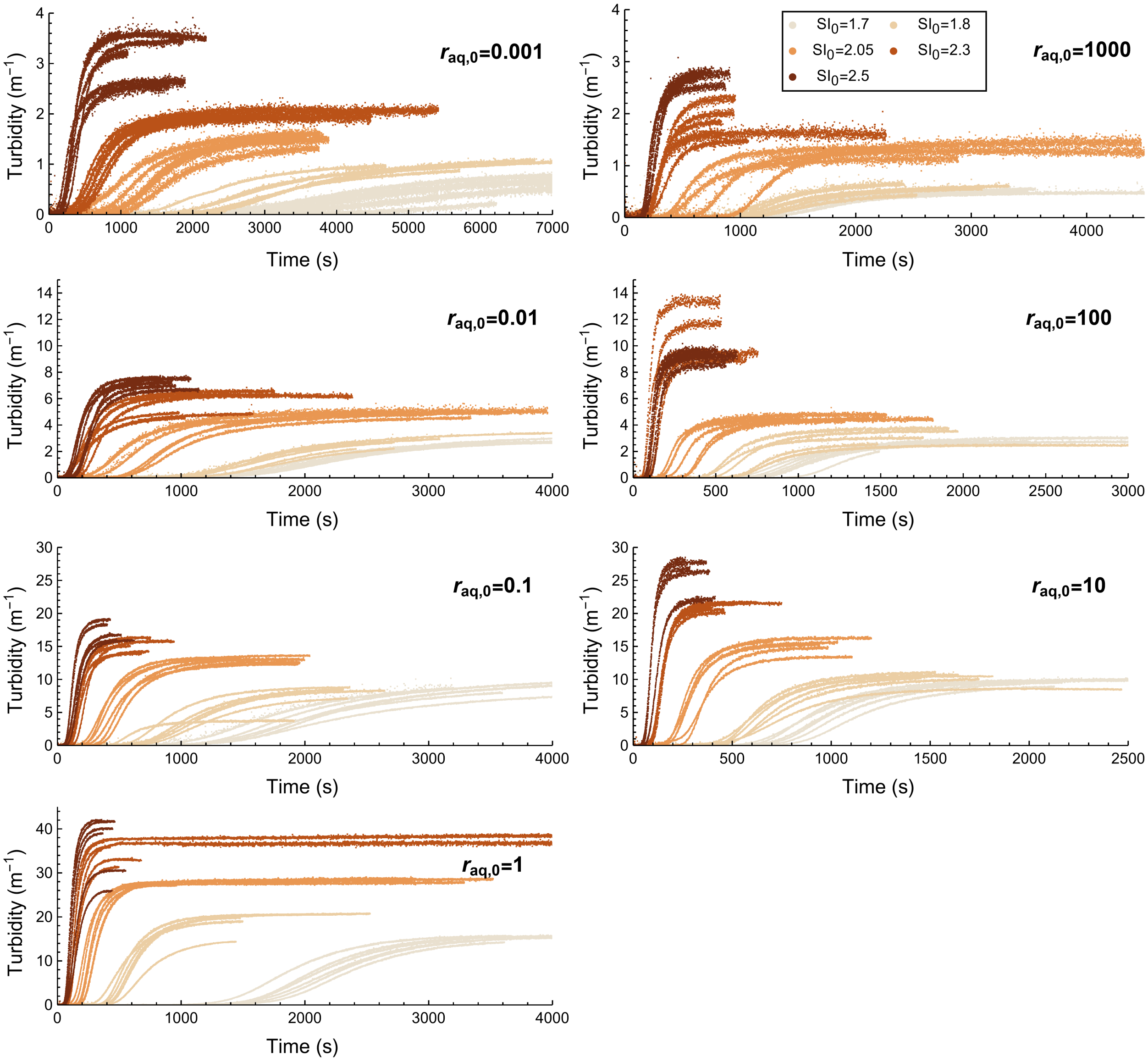

Figure S3: The turbidity is plotted over time for SI_0=1.7, 1.8, 2.05, 2.3, and 2.5 in a separate graph for $r_{aq,0}$ = 0.001, 0.01, 0.1, 1, 10, 100, and 1000.

6. Refitting of Measurements

The measurements were fitted similarly as before, but using a more extensive expression for G including an explicit r_{aq}-dependence as found with the semi-empirical rate model:

$$G = K\left(\sqrt{10^{SI}} - 1\right)^2 \left(\frac{1}{2r_{aq}e^{-1.694}} + \frac{r_{aq}e^{-1.694}}{2}\right)^{-0.239}, \tag{1}$$

This allows the r_{aq} to also change over time with:

$$r_{aq} = \frac{\gamma_{Ba^{2+}}\left([Ba^{2+}]_0 - \frac{4\pi R^3 n\rho}{3M}\right)}{\gamma_{SO_4^{2-}}\left([SO_4^{2-}]_0 - \frac{4\pi R^3 n\rho}{3M}\right)} \tag{2}$$

Below the main graphs from the modeling in the paper are shown following this different fit. On average the data fitted the model slightly better following the NRMSD, but the same main trends in the fit parameters and rates are observed. Note that the spread in K between all measurements indicates how well Eq. 1 describes the different measurements. Additionally, because the t_n values are slightly increased, 8 more measurements from $r_{aq,0} = 0.001$ and $r_{aq,0} = 0.01$ could be included in the analysis.

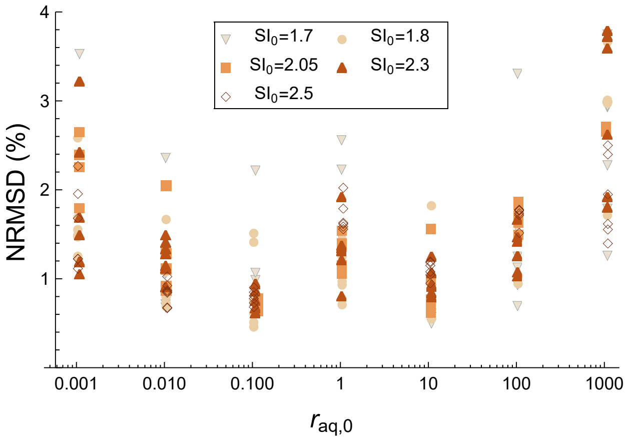

Figure S4: The normalised root-mean-squared-deviation (NRSMD) is indicated for the fits of all different measurements.

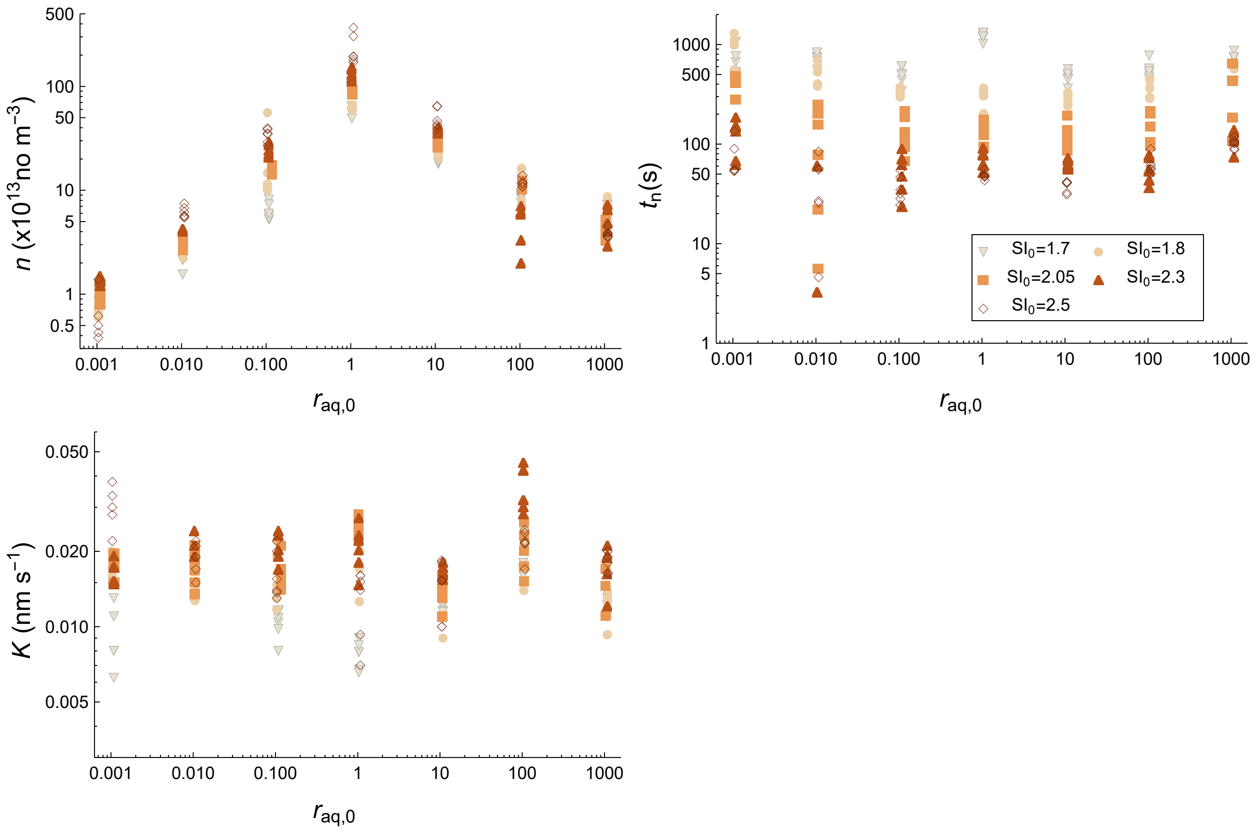

Figure S5: The number density of particles n, the nucleation time t_{n}, and the growth parameter K is plotted as a function of $r_{\mathrm{aq},0}$ for all SI_0.

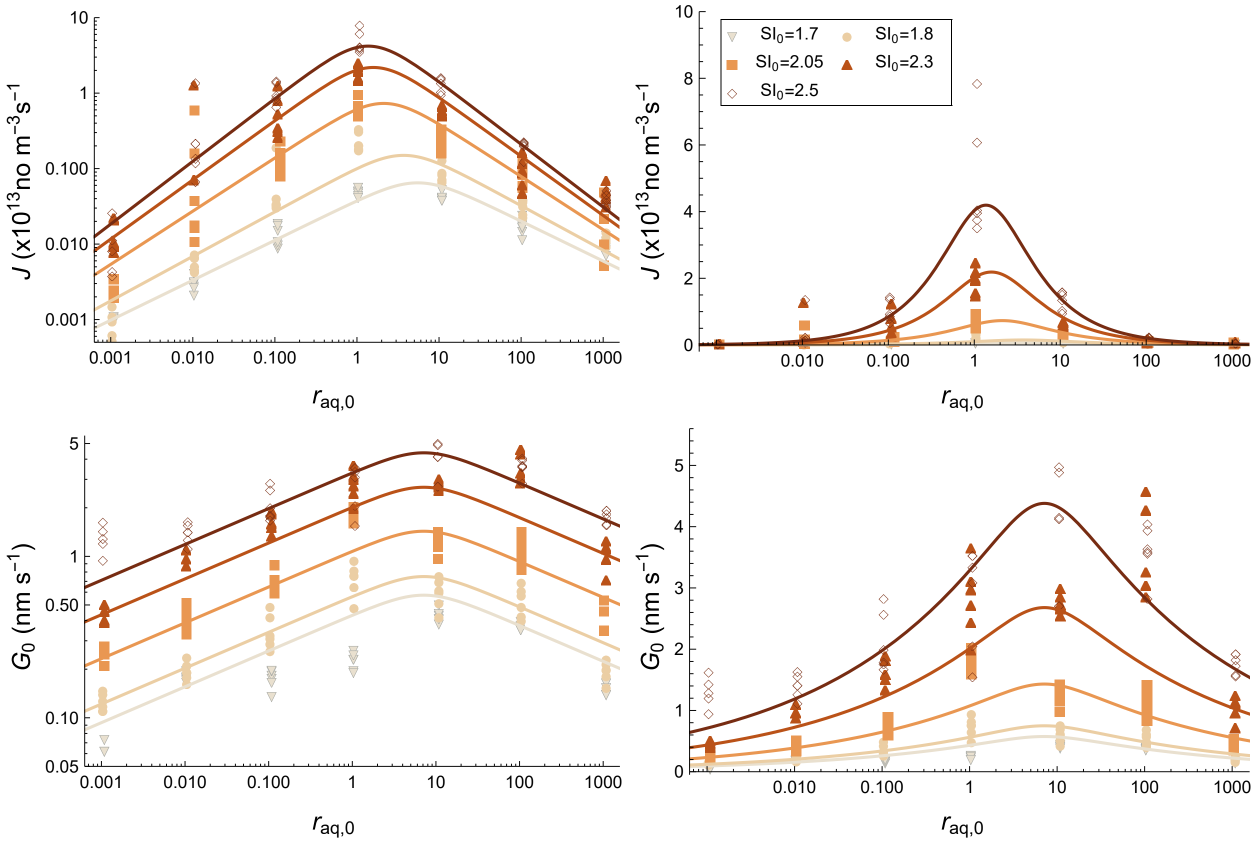

Figure S6: The nucleation rate J and initial growth rate G_0 are plotted as data points over $r_{\mathrm{aq},0}$. The curves represent the rate models.

7. Alternative plots of t_n

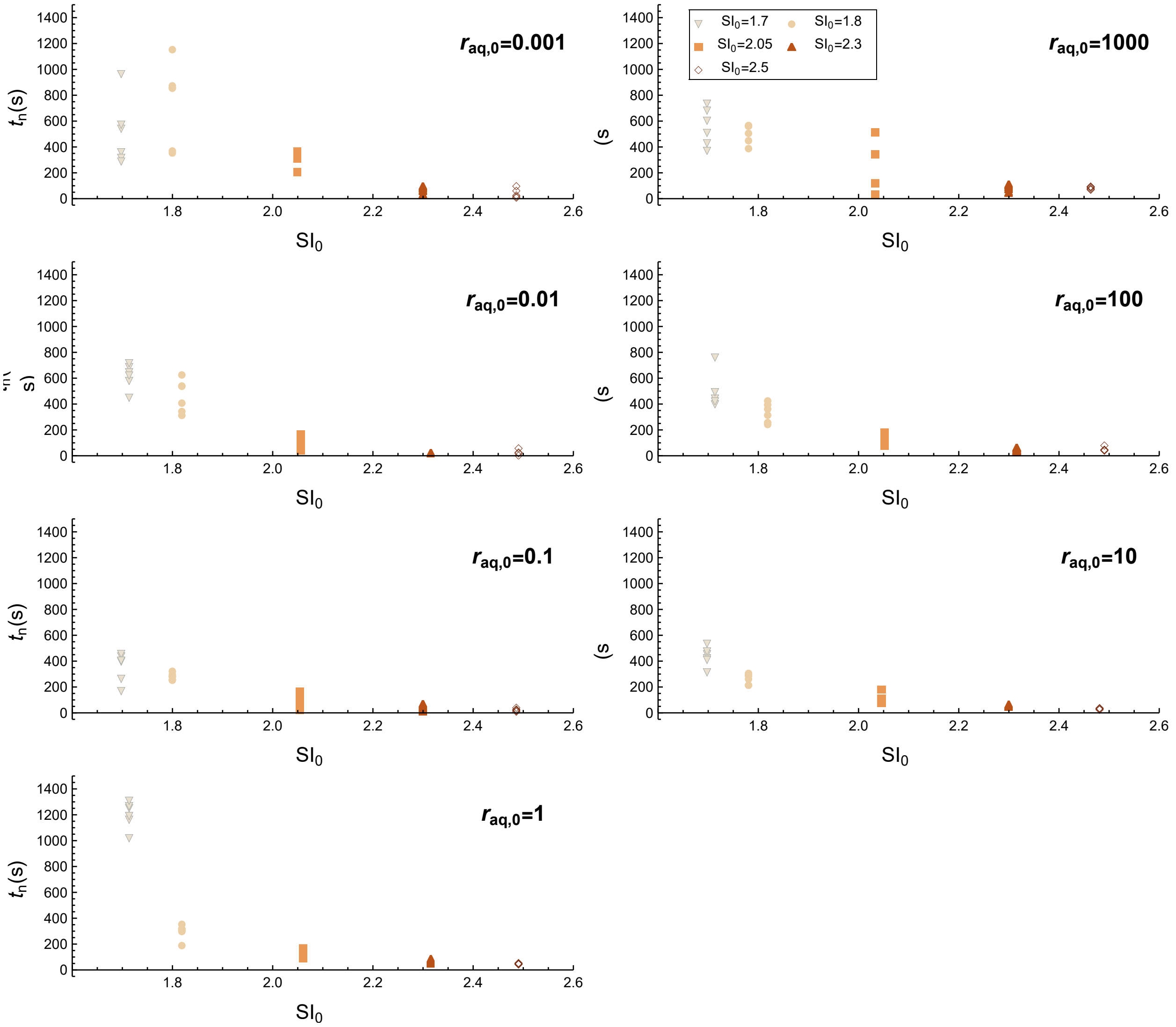

Figure S7: The nucleation time t_n is plotted over SI_0 in a separate graph for $r_{\mathrm{aq},0}$ = 0.001, 0.01, 0.1, 1, 10, 100, and 1000.

8. Example Fit with Aggregative Growth

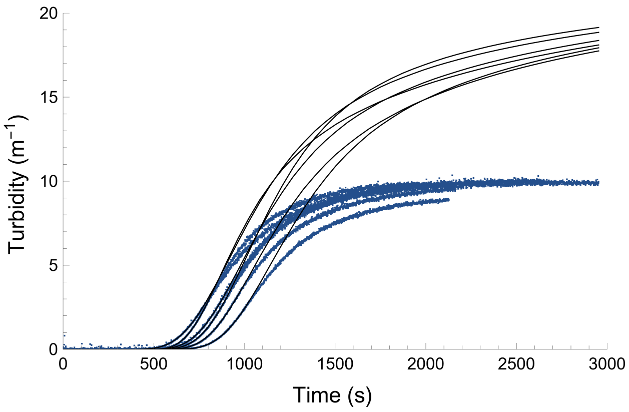

Figure S8: An example fit of the turbidity over time for $SI_0 = 1.7$ and $r_{aq,0} = 10$ using an alternative model including aggregative growth. The model is based on the same scattering and growth expressions as mentioned for Dai *et al.* in the 'Modeling' section, but using Eq. 6 for the primary particles size R_1 and the Brownian aggregation rate constant $k_a = 4k_BT/(3\mu)$ with the viscosity $\mu = 0.000\,891\,\mathrm{kg\,m^{-1}\,s^{-1}}$. As the model cannot describe the latter part of the data correctly, only the data up to roughly the inflection point was fitted.
